# Randomised Pharmacokinetic Trial of Rifabutin with Lopinavir/Ritonavir-Antiretroviral Therapy in Patients with HIV-Associated Tuberculosis in Vietnam

**DOI:** 10.1371/journal.pone.0084866

**Published:** 2014-01-22

**Authors:** Nguyen Thi Ngoc Lan, Nguyen Thi Nguyet Thu, Aurélie Barrail-Tran, Nguyen Hong Duc, Nguyen Ngoc Lan, Didier Laureillard, Truong Thi Xuan Lien, Laurence Borand, Catherine Quillet, Catherine Connolly, Dominique Lagarde, Alexander Pym, Christian Lienhardt, Nguyen Huy Dung, Anne-Marie Taburet, Anthony D Harries

**Affiliations:** 1 Pham Ngoc Thach Hospital, Ho Chi Minh City, Vietnam; 2 Pasteur Institute, Ho Chi Minh City, Vietnam; 3 Clinical Pharmacy Department, Bicêtre Hospital, Assistance-Publique Hôpitaux de Paris, Kremlin-Bicêtre, France; 4 ANRS, Ho Chi Minh City, Vietnam; 5 Epidemiology and Public Health Unit, Institut Pasteur du Cambodge, Phnom Penh, Cambodia; 6 Biostatistics Unit, Medical Research Council, Durban, South Africa; 7 The International Union Against Tuberculosis and Lung Diseases, Paris, France; 8 TB Research Unit, Medical Research Council, Durban, South Africa; 9 KwaZulu-Natal Research Institute for Tuberculosis and HIV (K-RITH), South Africa; 10 World Health Organization Stop Tuberculosis Program, Geneva, Switzerland; 11 Clinical Pharmacy and Pharmacokinetics Department (EA4123), University Paris Sud, Chatenay Malabry, France; 12 London School of Hygiene and Tropical Medicine, London, United Kingdom; University of Ottawa, Canada

## Abstract

**Background:**

Rifampicin and protease inhibitors are difficult to use concomitantly in patients with HIV-associated tuberculosis because of drug-drug interactions. Rifabutin has been proposed as an alternative rifamycin, but there is concern that the current recommended dose is suboptimal. The principal aim of this study was to compare bioavailability of two doses of rifabutin (150 mg three times per week and 150 mg daily) in patients with HIV-associated tuberculosis who initiated lopinavir/ritonavir-based antiretroviral therapy in Vietnam. Concentrations of lopinavir/ritonavir were also measured.

**Methods:**

This was a randomized, open-label, multi-dose, two-arm, cross-over trial, conducted in Vietnamese adults with HIV-associated tuberculosis in Ho Chi Minh City (Clinical trial registry number NCT00651066). Rifabutin pharmacokinetics were evaluated before and after the introduction of lopinavir/ritonavir -based antiretroviral therapy using patient randomization lists. Serial rifabutin and 25-O-desacetyl rifabutin concentrations were measured during a dose interval after 2 weeks of rifabutin 300 mg daily, after 3 weeks of rifabutin 150 mg daily with lopinavir/ritonavir and after 3 weeks of rifabutin 150 mg three times per week with lopinavir/ritonavir.

**Results:**

Sixteen and seventeen patients were respectively randomized to the two arms, and pharmacokinetic analysis carried out in 12 and 13 respectively. Rifabutin 150 mg daily with lopinavir/ritonavir was associated with a 32% mean increase in rifabutin average steady state concentration compared with rifabutin 300 mg alone. In contrast, the rifabutin average steady state concentration decreased by 44% when rifabutin was given at 150 mg three times per week with lopinavir/ritonavir. With both dosing regimens, 2 – 5 fold increases of the 25-O-desacetyl- rifabutin metabolite were observed when rifabutin was given with lopinavir/ritonavir compared with rifabutin alone. The different doses of rifabutin had no significant effect on lopinavir/ritonavir plasma concentrations.

**Conclusions:**

Based on these findings, rifabutin 150 mg daily may be preferred when co-administered with lopinavir/ritonavir in patients with HIV-associated tuberculosis.

**Trial Registration:**

ClinicalTrials.gov NCT00651066

## Introduction

In 2011, there were an estimated 34 million adults and children living globally with HIV/AIDS and an estimated 8.7 million new cases of tuberculosis: 1.1 million persons had HIV-associated tuberculosis and 430,000 persons with HIV-associated tuberculosis died [1], [2].

Since 2003, there has been a remarkable scale up of antiretroviral therapy with 8 million people estimated to be on therapy by the end of 2011 [1]. The most recent data show that 97% of adults and children on antiretroviral therapy are taking a first-line regimen, in general consisting of two nucleoside reverse transcriptase inhibitors and one non-nucleoside reverse transcriptase inhibitor [3]. The remainder is on a second-line regimen, usually consisting of a nucleoside reverse transcriptase inhibitor backbone and a protease inhibitor. The low number of patients on second-line treatment reflects the poor availability of viral load monitoring during antiretroviral therapy in resource-limited countries, and thus a limited ability to correctly diagnose treatment failure and switch patients accordingly to more effective therapy. With the development of point-of-care tests for viral load under the World Health Organization (WHO) new Treatment 2.0 initiative [4], and recommendations from the WHO that 12-monthly viral load monitoring should become the norm for monitoring antiretroviral therapy [5], it is likely that increasing numbers of patients will be identified with treatment failure and will need switching to a second-line regimen with a protease inhibitor. While this is a welcome move, this change will have implications for the care and treatment of patients with HIV-associated tuberculosis.

Observational studies have clearly shown that antiretroviral therapy improves the prognosis of patients with HIV-associated tuberculosis [6], and clinical trials have also established the importance of early initiation of antiretroviral therapy in reducing early mortality [7], [8], [9]. While first-line antiretroviral therapy using efavirenz is safe and effective when combined with rifampicin-based anti-tuberculosis treatment [10], there are challenges when it comes to using second-line regimens. The combination of rifampicin and protease inhibitors is problematic because rifampicin significantly reduces the bioavailability of all known protease inhibitors by 75% to 95% by induction of cytochrome 3A4 (CYP3A4) enzymes [11]. Attempts to overcome this adverse drug-drug interaction by either increasing the dose of the protease inhibitor or altering the dose of rifampicin have been thwarted by hepatotoxicity and other problems with tolerance [12], and such approaches are anyway incompatible with large-scale and decentralised public sector roll-out of ART.

Rifabutin is an attractive alternative to rifampicin as it is a less potent inducer of CYP3A4 [13], and the drug can safely be combined with ritonavir-boosted protease inhibitors without protease inhibitor dose adjustment. Rifabutin is recommended at a standard dose of 300 mg daily for the prophylaxis and treatment of *Mycobacterium avium* complex and for the treatment of drug-susceptible tuberculosis. Plasma concentrations of rifabutin are increased in the presence of protease inhibitors [11], and therefore dose adjustments are recommended. Guidelines from the Centers for Disease Control (CDC, Atlanta, USA) recommended in 1998 that the dose of rifabutin be reduced from 300 mg to 150 mg in the presence of a protease inhibitor [14], and the guidelines further recommended in 2004 that the dose be reduced to 150 mg three times a week (TPW) when used in combination with lopinavir/ritonavir (LPV/r) [15]. However, two recent reports have suggested that rifabutin given at a dose of 150 mg TPW in combination with LPV/r in patients with HIV-positive tuberculosis may result in inadequate rifamycin levels [16], [17]. Case reports of tuberculosis relapse in patients administered rifabutin 150 mg TPW with LPV/r [18] and further data showing that low rifamycin concentrations are associated with acquired rifamycin resistance in patients taking intermittent doses of rifabutin [19] all add to concerns that rifabutin given intermittently with protease inhibitor-based antiretroviral therapy is sub-optimal.

The present study was therefore undertaken with the primary objective of comparing the pharmacokinetic parameters of two doses of rifabutin (150 mg TPW and 150 mg daily) in patients with HIV-associated tuberculosis in Vietnam who initiated antiretroviral therapy with LPV/r. Secondary objectives were to investigate (i) the pharmacokinetics of LPV/r in combination with RBT, and (ii) the safety and toxicity of rifabutin in combination with antiretroviral therapy during the initial phase of anti-TB treatment.

## Methods

The protocol for this trial and supporting CONSORT checklist are available as supporting information; see Checklist S1 and Protocol S1.

### Ethics Statement

The study was approved by the Institutional Review Board and Ethical Review committee at Pham Ngoc Thach Hospital, the Health Department of Ho Chi Minh City and the Ministry of Health, Vietnam, as well as the Union Ethics Advisory Group of the International Union Against Tuberculosis and Lung Disease, Paris, France.

### Study design

This study was a randomized, open-label, multi-dose, two-arm, cross-over trial, conducted in Vietnamese patients with HIV-associated tuberculosis - Clinical trial registry number: NCT00651066.

### Study setting

The study was carried out in Pham Ngoc Thac Hospital, Ho Chi Minh, Vietnam, a tertiary care facility that has 800 beds and cares for TB patients, about 10% of whom have associated HIV-infection. In Vietnam, patients with suspected tuberculosis are investigated according to National Tuberculosis Guidelines [20] which are based on smear microscopy for acid-fast bacilli and chest radiography for those with pulmonary disease. Anti-tuberculosis treatment is given for 6 months and consists of a 2-months initial phase of rifampicin, isoniazid, pyrazinamide and ethambutol given as fixed dose combination tablets under direct observation, followed by 4-months continuation phase with rifampicin and isoniazid as fixed dose combination tablets. HIV testing is done at the time of tuberculosis registration [20], and HIV-positive patients are assessed with a CD4 lymphocyte count and started as soon as possible on a standard first-line antiretroviral therapy regimen - usually consisting of stavudine or zidovudine – lamivudine – efavirenz as a standard fixed dose combination.

### Patient recruitment

Study patients were adults aged 18 – 65 years, HIV-positive, with a CD4 count less than or equal to 250 cells/µL and with newly diagnosed tuberculosis. Eligibility requirements included:- provision of written informed consent; having a firm home address that was readily accessible; if female, having a negative pregnancy test on day of enrolment; having a diagnosis of pulmonary tuberculosis confirmed by smear microscopy, culture or a chest radiograph compatible with active tuberculosis and associated with a typical clinical history and two negative sputum smears; no previous history of antiretroviral therapy; weight ≥40 kg; a Karnofsky score Q ≥80%; no grade 3 or 4 clinical or laboratory findings according to Division of AIDS tables [21]. Patients with the following conditions were excluded from the trial: a previous episode of tuberculosis within the last 12 months, a history of prior treatment for multi-drug resistant tuberculosis (resistant to at least rifampicin and isoniazid), concomitant opportunistic infection requiring additional anti-microbial treatment, a formal contraindication to any trial medication including hypersensitivity, diabetes mellitus requiring treatment, recreational drug or alcohol abuse, mental illness, total neutrophil count <1200 cells/L, hemoglobin <6.8 g/dL, or liver function tests > grade 2 (according to DMID tables). Pregnant or lactating women or women unwilling to use appropriate contraception were also excluded. Patients were recruited to the study between 27 September 2011 and 27 March 2012.

### Randomization

Patients were randomized to receive one of two individual treatment arms on the day of enrolment. Randomization lists were produced prior to the start of the trial by the Medical Research Council in South Africa (ratio 1∶1, mixed size blocks). The clinical research team in Vietnam used pre-prepared envelopes in chronological order, indicating to which treatment arm the patient should be assigned.

### Treatments under study

**Figure 1:**
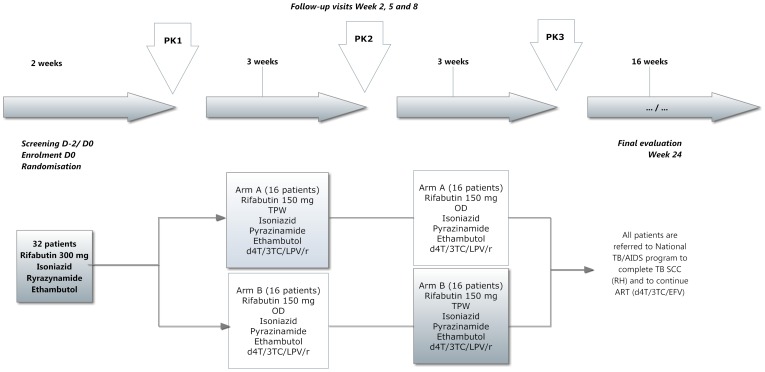
Timeline of the pharmacokinetic trial of rifabutin with antiretroviral treatment in HIV-infected patients with tuberculosis in Vietnam. PK =  pharmacokinetic analyses; TPW =  three times per week; OD  =  once per Day; d4T =  stavudine; 3TC  =  lamivudine; LPV/r  =  lopinavir/ritonavir; TB  =  tuberculosis; SCC  =  short course chemotherapy; RH =  rifampicin and isoniazid; ART  =  antiretroviral therapy; EFV  =  efavirenz

The detailed planned trial timeline describing the intended allocation of treatments in the two arms of the trial in relation to the initial and continuation phases of anti-tuberculosis treatment and randomization is shown in **Figure 1**. As no wash out period was possible, all pharmacokinetic parameters were estimated at steady state, at least 2 weeks after initiation of rifabutin treatment or with the new dosing regimens. At enrolment into the trial, patients were started on rifabutin 300 mg once a day (OD), in combination with standard doses of isoniazid, pyrazinamide and ethambutol. After two weeks (representing the first 2 weeks of the initial phase of treatment) the first pharmacokinetic study (PK1) was done. Patients were continued on the same anti-tuberculosis treatment and at two weeks from the start of anti-tuberculosis treatment were started on antiretroviral therapy with stavudine-lamivudine-lopinavir/ritonavir (d4T/3TC/LPV/r – standard doses of stavudine 30 mg/lamivudine 150 mg/lopinavir/ritonavir 400 mg/100 mg – taken twice daily) and randomized to one of two arms:- Arm A =  Rifabutin 150 mg TPW or Arm B =  Rifabutin 150 mg OD. After a further three weeks, the second PK (PK2) study was done and the treatments crossed-over: patients on the “A” dose of Rifabutin were switched to the “B” dose and vice versa. Patients remained on these doses along with isoniazid, pyrazinamide and ethambutol and antiretroviral therapy for a further three weeks and the third PK study (PK3) done. After PK3, the patients stopped rifabutin and started the continuation phase of anti-tuberculosis treatment with rifampicin and isoniazid under the care of the National Tuberculosis Program. They were also referred to the National AIDS Program to be treated according to standard care with stavudine/lamivudine/efavirenz. Patients were followed up to the end of anti-tuberculosis treatment for another 16 weeks. Physical examinations and laboratory investigations were done at every PK study.

Laboratoires SERB supplied rifabutin 150 mg capsules for oral administration (Ansatipine 150 mg, Pfizer) and the new film-coated tablet formulation of LPV/r, Aluvia® was purchased from Abbott Laboratories (USA).

### Sample size

Based on the area under the curve (AUC_0-24_) for rifabutin determined in previous studies [19], it was estimated that a sample size of 12 participants had a power of 80% to detect a 20% relative change between the geometric means of the AUC_0-24_ for the participants taking rifabutin without antiretroviral therapy and the AUC_0-24_ for the participants taking rifabutin when combined with antiretroviral therapy. To provide a target of 12 evaluable patients in each arm, because patients with low CD4 cell counts recruited into the study might experience high mortality and morbidity resulting in a high attrition rate, it was decided that 32 patients should be enrolled (16 in each arm).

### Pharmacokinetic (PK) sampling and drug analysis

All patients were admitted to the Clinical Trial Unit facility the night before each PK study and were fasted from midnight. On the morning of the PK sampling day, serial blood samples were obtained. The first blood sample (0 h) was drawn prior to administration of study drugs and a standard hospital breakfast was served exactly two hours (2 h) after drug ingestion. Subsequent bloods were drawn at 2, 3, 4, 5, 6, 8, 12, 24 and 48 h (in the case of intermittent RBT dosing) after drug ingestion. The samples were placed on ice immediately and centrifuged at 3000 rpm at 4°C for 10 minutes within 30 minutes of collection. Separated plasma was transferred to polypropylene tubes and stored immediately at -70°C until analysis. The drug assays for RBT and its metabolite (25-O-desacetylrifabutin) as well as lopinavir and ritonavir are described in the following section [22].

#### Drug analyses for rifabutin, 25-O-desacetylrifabutin, lopinavir and ritonavir

Rifabutin and 25-O-desacetylrifabutin were analyzed simultaneously with a validated HPLC assay. Rifabutin and 25-O-desacetylrifabutin standards were kindly provided by Pfizer. In brief, after addition of medazepam as internal standard both chemicals were extracted from 0.2 mL of plasma with a hexane/dichloromethane solution (6/4 v/v). After vortex and centrifugation, the organic phase was evaporated to dryness. Dry residue was reconstituted with 100 µL of mobile phase constituted of [Phosphate mono potassic dihydrogen solution 0.05 M, pH = 3,85]/acetonitrile: 600/400 (v/v). 50 µL is injected onto the Eclipse XDB RP-C18, 150×4, 6 mm, 5 µm – Agilent column. The spectrophotometer for UV detection was set at 272 nm. Lower limits of detection were 12.5 ng/mL and 6.25 ng/mL for rifabutin and desacetyl rifabutin respectively. Linearity of standard curves was demonstrated up to 500 ng/mL and 250 ng/mL for rifabutin and desacetyl rifabutin respectively. Variability of day to day quality controls inserted in each analytical run was lower than 9% for median and high concentrations and lower than 15% for low concentrations. The accuracies (as % of nominal value) for rifabutin and 25-O-desacetylrifabutin were between 97% and 106% at low, medium and high QC levels during inter-run validation.

Plasma lopinavir and ritonavir concentrations were quantified by a validated reverse phase HPLC method as described elsewhere [22] with slight modifications. The limit of quantification was 50 ng/mL for lopinavir and ritonavir. Linearity of standard curves was demonstrated up to 10000 ng/mL and 5000 ng/mL for lopinavir and ritonavir respectively. Variability of day to day quality controls inserted in each analytical run was lower than 6% for median and high concentrations and lower than 9% for low concentration. The accuracies (as % of nominal value) for lopinavir and ritonavir were between 98% and 110% at low, medium and high QC levels during inter-run validation. Asqualab quality controls (France) were inserted in each lopinavir and ritonavir analytical runs.

### Pharmacokinetic analysis

The main pharmacokinetic measures for rifabutin, 25-O-desacetylrifabutin and lopinavir were derived by non-compartmental analysis using WinNonLin software (Pharsight, USA). The peak concentration (C_max_), and time to C_max_ (T_max_) were obtained directly from the concentration-time profiles. Drug concentrations at the end of a dosing interval were reported as C_min_ and pre-dose concentrations on the day of pharmacokinetic evaluation reported as C_0_. The steady-state AUC (AUCτ) during a dosing interval τ 24 hours or 48 hours for rifabutin and 12 hours for lopinavir/ritonavir were calculated for each drug by the linear up/log down trapezoidal method. As an index of exposure during a dosing interval, the average concentration at steady state (Cave) was calculated for rifabutin and its metabolite as Cave = AUCτ/τ where τ is the dosing interval. The metabolite ratio was calculated as the ratio of metabolite to parent drug AUCs.

### Analysis and statistics

The steady state pharmacokinetics of rifabutin and 25-O-desacetylrifabutin were determined at each of the three pharmacokinetic evaluations and the pharmacokinetics of lopinavir were determined after the second and third pharmacokinetic evaluations. In order to identify an effect of sequence randomization on the pharmacokinetic measures, a linear mixed effects regression model using baseline dose considered as reference (rifabutin 300 mg daily) as a covariate was applied. As no sequence or day effect was found, the drug groups were pooled and dose levels were compared. Rifabutin parameters for assessing the interaction when combined with LPV/r were Cmax, C0, and Cave. These parameters were logarithmically (log) transformed and a linear mixed model fit was used which included treatment, period and sequence as fixed effects and the patient as a random effect. Ninety percent confidence intervals (90% CIs) for the difference in mean log-transformed (log) PK parameters for a particular rifabutin combination therapy (150 mg OD or 150 mg TPW) compared to rifabutin monotherapy (300 mg OD) were calculated. These differences in mean log PK parameters and 90% CIs were back transformed and presented in their original units as geometric means and 90% CIs. The geometric mean ratio presented in **Table 1** can be interpreted as a relative change (either fold or percentage) in geometric mean PK parameters for a particular combination therapy compared to rifabutin monotherapy The rifabutin regimen combined with LPV/r was deemed equivalent to rifabutin alone when the 90% CI for the ratio fell within the equivalence interval of 0.80% to 1.25%. Baseline and final viral loads and CD4^+^ counts were compared using t-tests. A p value <0.05 was considered significant.

**Table 1:** Geometric mean ratios and 90% confidence intervals of rifabutin and 25-O desacetyl rifabutin parameters measured for rifabutin plus lopinavir/ritonavir and rifabutin.

	Rifabutin		25-O desacetyl rifabutin	
	150 mg OD	150 mg TPW	150 mg OD	150 mg TPW
Cmax	0.88 (0.75;1.04)	0.65 (0.51;0.83)	2.58 (2.04;3.25)	1.57 (1.21;2.03)
C0	2.61 (2.13;3.19)	0.94 (0.73;1.21)	11.49 (8.21;16.09)	4.35 (3.14;6.02)
AUCτ	1.32 (1.16;1.51)	1.12 (0.92;1.37)	5.13 (3.94; 6.69)	4.74 (3.59; 6.25)
Cave	1.32 (1.16;1.51)	0.56 (0.46;0.69)	5.13 (3.94; 6.69)	2.36 (1.79;3.12)

GMR  =  geometric mean ratio; OD  =  once daily; TPW  =  three times per week; LPV/r  =  lopinavir/ritonavir

AUCτ is AUC_24h_ for OD and AUC_48h_ for TPW. Cave is average concentration at steady state calculated as AUCτ/τ, where τ is the dosing interval for RBT, 24 or 48 h

## Results

### Patient flow chart

**Figure 2:**
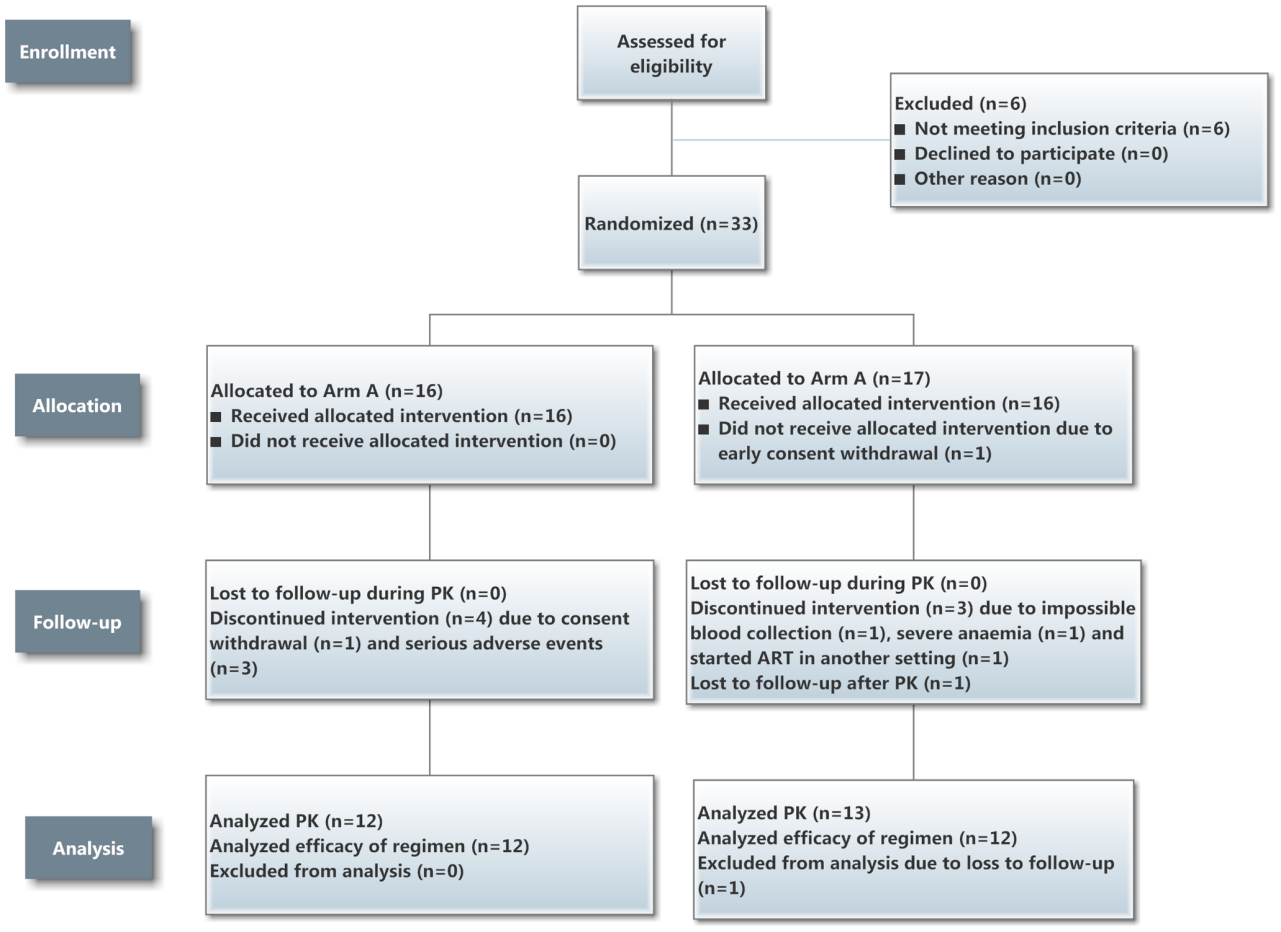
Patient Flow Chart for the trial. PK =  pharmacokinetic analyses

Thirty nine patients were assessed for eligibility for the trial, with **Figure 2** showing the numbers randomized, allocated to interventions in Arm A and B, followed-up and subsequently having blood measurements for pharmacokinetic analysis. Altogether 33 patients were randomized. One patient in Arm B did not receive the allocated intervention due to early consent withdrawal, leaving 16 to receive the allocated intervention in each arm. In Arm A, four patients discontinued the intervention – one due to consent withdrawal and three due to serious adverse events (one with cryptococcal meningitis and two with hepatitis in the first two months of treatment. In Arm B, three patients discontinued the intervention – one due to impossible venous puncture as a result of being a previous intravenous drug user, one due to severe anaemia and one starting antiretroviral therapy in another setting before the first PK analysis. Thus, 25 patients underwent the three pharmacokinetic visits (12 in Arm A and 13 in Arm B). One patient in Arm B was lost-to –follow-up after the pharmacokinetic analysis, leaving 24 to complete anti-tuberculosis treatment.

### Patient demographic and clinical characteristics

**Table 2** shows the baseline characteristics of the 33 patients and the 25 who completed the PK analyses. Of the 25 patients who completed PK analyses, all had a Karnosky score of 90. There were 14 who had smear-positive pulmonary tuberculosis and 11 who had smear-negative pulmonary tuberculosis. In 18 of these patients, *Mycobacterium tuberculosis* was isolated from sputum, and in one of these patients resistance to rifampicin and isoniazid was diagnosed after completing the initial phase of treatment. Patients were on no other drugs except for anti-tuberculosis drugs and ARV drugs.

**Table 2:** Base-line characteristics of HIV-infected tuberculosis patients in Vietnam.

	Median (IQR) N = 33 enrolled patients		Median (IQR) N = 25 completing PK studies	
Age in years	32.7	(28.6 – 35.1)	32.7	(27.6 – 35.1)
Male (%)	28	(85%)	21	(84%)
Weight in Kg	50.4	(45.5 – 54.50)	49	(44.50 – 53.50)
BMI (1)	18.6	(17.31 – 20.52)	18.0	(17.26 – 19.92)
CD4 Count cells/mm^3^	65	(23 – 135)	65	(26 – 126)
Plasma HIV-RNA logcopies/mL (2)	5.79 (3)	(5.26 – 6.22)	5.87	(5.32 – 6.18)

IQR – inter-quartile; PK  =  pharmacokinetic; BMI  =  body mass index;

(1) 16/33 or 13/25 patients were underweight (BMI<18.5) and 17/33 or 12/25 were normal (BMI>18.5- 25.6)

(2) Measured at the second visit (Day 14) before antiretroviral therapy initiation

(3) N = 30

### Rifabutin and 25-O-desacetylrifabutin pharmacokinetics

**Figure 3:**
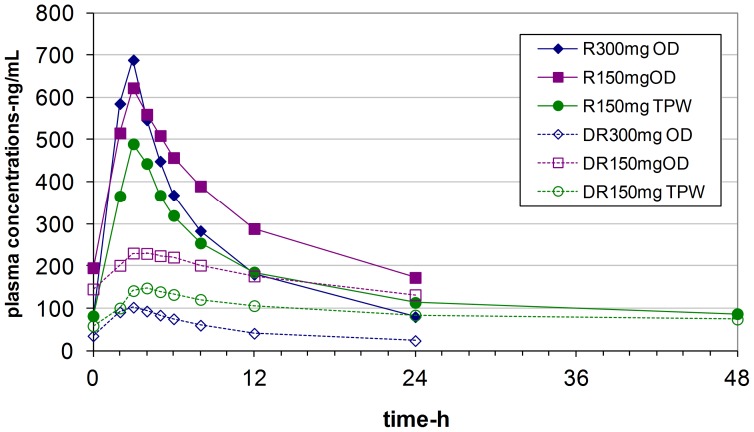
Plasma concentrations plotted against time for rifabutin (R) and 25 desacetyl rifabutin (D) in relation to whether rifabutin was administered alone (300 mg) or combined with lopinavir/ritonavir at 150 mg OD or TPW. OD  =  once daily; TPW  = three times per week

Plots of mean concentrations of rifabutin and its desacetyl metabolite against time are shown in **Figure 3**. Plasma concentrations of 25-O-desacetylrifabutin were always lower than those of rifabutin concentrations at whatever dose of rifabutin used. Concentrations of rifabutin and 25-O-desacetylrifabutin were higher when rifabutin was combined with LPV/r compared with when it was administered alone, and higher concentrations were observed with the 150 mg OD dose compared with the 150 mg TPW dose. Pharmacokinetic parameters of rifabutin and 25-O deacetyl rifabutin are compared in **Table 3**. Morning pre-dose trough (C0) concentrations were higher when rifabutin was administered OD with LPV/r compared with TPW. The peak concentrations (Cmax) and the area under the curve (AUCτ) were similar whatever the dosing regimen, although slightly higher levels were observed with rifabutin 150 mg OD. Overall, a large inter-individual variability in pharmacokinetic parameters of rifabutin was observed. Individual metabolic ratios (25-O-desacetylrifabutin/rifabutin) showed a similar pattern with higher ratios observed when rifabutin was combined with LPV/r (medians 0.57 and 0.64 for 150 mg OD and 150 mg TPW respectively compared with when rifabutin was used alone with a median of 0.13) The geometric mean ratios of rifabutin and 25-O-desacetylrifabutin are shown in **Table 1**. When rifabutin 150 mg OD was combined with lopinavir/ritonavir, Cmax was only slightly lower than when rifabutin was administered alone, and a 2 to 3-fold increase in trough concentrations was observed. With the TPW dosing, a 35% decrease in Cmax was observed although pre-dose concentrations were close to meeting equivalence with rifabutin monotherapy. Assuming that the average concentration at steady state (Cave) represents plasma exposure, the two tested rifabutin dosing regimens combined with lopinavir/ritonavir failed to show bioequivalence. Only rifabutin at 150 mg OD with LPV/r led to a significantly 32% higher rifabutin Cave compared with when it was administered alone. Rifabutin Cave reached after the TPW regimen was lower compared with rifabutin alone. A large increase in 25-O desacetyl rifabutin concentrations was observed when rifabutin was co-administered with lopinavir/ritonavir. Cave was increased by a factor of two to five with the OD and TPW dosing respectively.

**Table 3:** Pharmacokinetic parameters of rifabutin and 25-O desacetyl rifabutin.

	Rifabutin			25-O desacetyl rifabutin		
	Alone	with lopinavir/ritonavir		Alone	with lopinavir/ritonavir	
	300 mg OD	150 mg OD	150 mg TPW	300 mg OD	150 mg OD	150 mg TPW
Cmax ng/mL	792 (344 – 1105)	671 (246 – 1146)	544 (55 – 964)	80 (25 - 595)	216 (94 – 535)	142 (31 – 308)
Tmax h	3 (2 – 4)	3 (2 – 5)	3 (0 – 5)	3 (0 – 5)	4 (2 – 8)	4 (2 – 6)
C0 ng/mL	74 (13 – 161)	180 (121 – 310)	70 (25 – 413)	10 (0 – 595)	137 (48 – 334)	54 (14 – 118)
Cmin ng/mL n = 25	79 (13 – 170)	169 (71 – 320)	NA	6 (6 – 329)	115 (59 – 253)	NA
Cmin ng/mL n = 15	61 (13 – 118)	161 (71 – 289)	54 (13 – 414)	6 (0 – 33)	114 (73 – 253)	67 (11 – 214)
AUCτ ng.h/mL	5640 (2715–8876)	7292 (3524–12514)	7344 (1426–10896)	697 (245–10250)	4127 (1769–8616)	3807 (872–7628)
Cave ng/mL	235 (113–370)	304 (147–521)	153 (30–227)	29 (10–427)	79 (18–159)	172 (74–359)

Data are presented as medians with the range in parenthesis

OD – once daily; TPW - three times per week; Cmax -peak concentration; Tmax - time to reach peak concentration; C0 - concentration at time 0; Cmin -concentration at the end of a dosing interval (24 h or 48 h);NA non available, Cmin 48 h post dosing non available in 10 patients; AUCτ – area under the curve during a dosing interval τ, τ is 24 h for OD dosing and 48 h for TPW. Cave – average concentration (AUCτ/τ).

### Lopinavir and ritonavir pharmacokinetics

The median trough and peak concentrations (C0 and Cmax) of lopinavir and ritonavir with rifabutin 150 mg OD and 150 mg TPW are shown in **Table 4**. There was again wide inter-individual variation in individual trough concentrations, which were similar across rifabutin dose regimens. The study design did not allow comparison of lopinavir and ritonavir concentrations when combined with and without rifabutin.

**Table 4:** Lopinavir and ritonavir pharmacokinetic parameters.

	Lopinavir			Ritonavir		
	RBT OD	RBT TPW	All patients	RBT OD	RBT TPW	All patients
Cmax – ng/ml	15439 (7540–34490)	18154 (7803–39550)	16 065 (7540–39550)	777 (332–1587)	816 (405–2484)	815 (32–2484)
C0 – ng/mL	9155 (399–27567)	8014 (50–31171)	8739 (<50– 31171)	314 (25– 569)	257 (25–680)	303 (25–680)

Data are presented as medians with the range in parenthesis

RBT  =  rifabutin; OD  =  once daily; TPW  =  three times per week; Cmax  =  peak concentration; C0  =  trough concentration. All patients: data pooled whatever the RBT dosing 150 mg OD or 150 mg TPW.

### Adverse events

The 33 enrolled patients had a total of 124 adverse events (all grades together). Eighty percent of the adverse events were low grade (grades 1 and 2). Hepatic events with raised levels of liver enzymes were the commonest adverse events with 56 events occurring in 25 patients. Of these, seven were grade 3 or 4. Of these hepatic events, 33 occurred in the first 2 months and 23 after rifabutin was stopped; their average duration was more than 66 days. There was one case of IRIS (immune reconstitution inflammatory syndrome) grade 3 and no uveitis. There were 4 cases of neutropenia but only one that was grade 3 and none that was grade 4. Serious adverse events are shown in **Table 5**.

**Table 5:** Serious adverse events in patients who completed all pharmacokinetic assessments (N = 25) and in patients who did not complete the assessments (N = 8).

9 serious adverse events seen in 7 patients who completed the study	• Hernia of an intervertebral disc
	• Severe anaemia
	• Immune reconstitution inflammatory syndrome
	• Cholestatic hepatitis
	• MDR-TB causing bilateral lymphadenopathy
	• Unidentified abdominal mass
	• Polyarthralgia (2 occurrences)
	• *Pneumocystis carinii (jerovici)* pneumonia
5 serious adverse events in 5 patients who did not complete the study	• Acute hepatitis followed by death
	• Severe hepatitis and recovered
	• Polyarthritis
	• Cryptococcal meningitis
	• Severe anaemia and respiratory failure followed by death

### Response to treatment

Among the 24 patients who completed anti-tuberculosis treatment with all PK visits scheduled, 22 (92%) had negative cultures for *Mycobacterium tuberculosis* and 2 had positive cultures (one patient was sputum smear negative but had drug-resistant TB with resistance to isoniazid and rifampicin and one patient was sputum-smear positive for acid-fast bacilli with the culture indicating non-tuberculous mycobacteria). For the 24 study patients, the median (IQR) increase in CD4 cells/mm^3^ was 127 (64–170) – there were two patients who had a decrease from 229 to 188 and 223 to 219 cells/mm^3^. Plasma HIV-RNA was undetectable (<250 copies/mL) for 19 (79%) of the 24 study completers. Five patients had a detectable HIV-RNA without any resistance mutations at HIV genotyping.

## Discussion

This is one of the first studies to investigate whether doses of rifabutin at 150 mg once daily or 150 mg three times per week are suitable in combination with the tablet formulation of LPV/r in an antiretroviral therapy regimen in the treatment of patients with HIV-associated tuberculosis. The main findings were that peak concentrations (C_max_) and the area under the curve (AUCτ) of the drugs were in the same range, regardless of the dose used. There was a significant and almost one third higher average concentration at steady state of rifabutin when used with LPV/r at a dose of 150 mg daily compared with 300 mg alone. The intermittent dosing of rifabutin co-administered with LPV/r led to a lower average concentration compared with 300 mg alone, although pre-dose concentrations remained in the same range. The different doses of rifabutin had no significant effect on the concentrations of lopinavir or ritonavir. Although there were a large number of recorded adverse effects, these were largely low grade and mainly related to an increase in serum liver enzyme levels. Of the 24 patients who completed the pharmacokinetic studies and who completed six months of anti-tuberculosis treatment, over 90% had negative *Mycobacterium tuberculosis* cultures, all but two patients had a measurable increase in CD4 cell counts and over 70% of patients had undetectable viral loads.

There have been previous studies assessing the pharmacokinetic interaction of rifabutin with ritonavir-boosted HIV protease inhibitors (fosamprenavir, darunavir, atazanavir and saquinavir [23], [24], [25], [26]. All these studies were conducted in healthy volunteers with various rifabutin dosing regimens, 150 mg or 300 mg OD when rifabutin was administered alone and 150 mg once every other day, twice weekly or every 3 days when combined with a protease inhibitor. All these studies showed that when the rifabutin dose was reduced in the presence of a potent drug metabolizing enzyme inhibitor (namely a protease inhibitor) this led to unchanged or moderate increases in rifabutin concentrations and a large increase in rifabutin metabolite concentrations. Interestingly, the steady state concentrations seen with the daily dose of rifabutin in the absence of antiretroviral therapy were in the same range as or somewhat lower than those described in our Vietnamese population [25], [26]. There have not been previous published studies assessing these drug-drug interactions when using the tablet formulation of LPV/r (Aluvia), which is now the most widely used protease inhibitor formulation in global HIV programs due to its heat stable properties [22].

A different version of the current study was carried out in South Africa from 2008 to 2010, in which the start of antiretroviral therapy was at 10 weeks after the start of anti-tuberculosis treatment when the patient was in the continuation phase on rifabutin and isoniazid [27]. In the South African study, it was found that the peak concentrations of rifabutin were significantly reduced in patients taking rifabutin three times a week, and, furthermore, over 85% of patients on the intermittent dose had areas under the curve less than 4.5 µg.h/mL, levels which have previously been associated with acquired rifamycin resistance. Interestingly, rifabutin concentrations were higher in our Vietnamese population, and only one patient had a Cmax less than 0.3 µg/mL on the 150 mg TPW regimen. The AUCτ of rifabutin during the dosing interval were higher than those measured in the South African population (median levels for the 300 mg OD dose were 5640 ng.h/mL in Vietnam compared with 3053 ng.h/mL in South Africa). These differences may be due to ethnic differences or other differences in the two populations – for example, the median body mass index was 18 in Vietnam and 23 in South Africa. In both Vietnam and South Africa, LPV/r led to a significant increase in rifabutin concentrations with the 150 mg OD regimen and a decrease in rifabutin concentrations with the 150 mg TPW regimen. As a consequence of higher rifabutin concentrations in Vietnam, only one patient had an AUCτless than 4.5 µg.h/mL on the 150 mg OD regimen compared with four on the 150 mg TPW and six with the 300 mg OD regimen.

Although the study was not designed to compare lopinavir and ritonavir concentrations on and off anti-tuberculosis treatment, trough lopinavir concentrations were higher than those observed in previous studies [22]. There have been reports for example of increased lopinavir concentrations on rifabutin which have decreased once rifabutin was discontinued [28]. Importantly in our study, the findings showed that lopinavir/ritonavir concentrations were not reduced during rifabutin therapy.

It was initially planned that the same study design run in South Africa would be implemented in Vietnam. However, for various reasons implementation of the Vietnam study was delayed, and by the time patients were being recruited, the WHO had released their 2010 Guidelines for ART, recommending that antiretroviral therapy should start between 2 – 8 weeks after the start of anti-tuberculosis treatment [29]. Investigators in the Vietnam study felt that the Vietnam study protocol starting antiretroviral therapy at 10 weeks was in conflict with recommended international best practice [30]. The trial was stopped and an amended study protocol with patients starting antiretroviral therapy two weeks after start of anti-tuberculosis treatment as presented in this paper was developed and implemented instead.

Although we were only able to study the effect of rifabutin with LPV/r in the intensive phase of anti-tuberculosis treatment, we continued with the cross-over design to ensure that if there was any sequence effect of the different rifabutin doses on pharmacokinetic measures this would be identified. In the event, no sequence or day effect was found, and the drug groups could therefore be pooled and dose levels compared. There is still controversy over whether Cmax or AUCτ is the best pharmacodynamic measure for rifamycins in general. Some studies on guinea pigs have found Cmax to be the critical pharmacokinetic parameter [31], [32], while other studies on mice and using hollow fibers have found that AUCτ is a superior parameter [33], [34]. For these reasons, both parameters were measured and reported on in this study, showing that rifabutin and 25-O-desacetylrifabutin levels were higher using the daily dose with LPV/r than without, and with the average concentration at a steady state being one third higher using 150 mg daily and 40% lower using 150 mg TPW compared with rifabutin alone. A limitation of this study is that we cannot provide answers about the toxicity or efficacy of single dose rifabutin, and a more formal clinical trial is warranted to determine whether daily rifabutin with an increase in rifabutin concentrations is associated with improved efficacy and acceptable adverse effects.

In conclusion, this study supports the use of rifabutin given at a dose of 150 mg once daily when combined with LPV/r based antiretroviral therapy, at least in patients with a low body mass index. It is not possible to generalize the results of this study to other ethnic groups outside of South-East Asia who may differ in their body mass index and in the way in which they metabolize drugs. The WHO Guidelines for the treatment of HIV-associated tuberculosis [10] recommend that treatment is given daily throughout the intensive and continuation phases of anti-tuberculosis treatment. Giving rifabutin as a daily dose is in line with these recommendations. This would facilitate the important programmatic issue of combining rifabutin with other anti-tuberculosis medications as a fixed-dose combination pill to be taken on a daily basis, a necessary measure if the results of this and other research are going to reach patients being managed routinely within general health service care.

## Supporting Information

Checklist S1 **CONSORT Checklist.**
(DOC)

Protocol S1 **Trial protocol.**
(PDF)
