# Supplementary material for: Randomised Pharmacokinetic Trial of Rifabutin with Lopinavir/Ritonavir-Antiretroviral Therapy in Patients with HIV-Associated Tuberculosis in Vietnam
Source: PLoS One. 2014 Jan 22;9(1):e84866. doi: 10.1371/journal.pone.0084866 (PMC3898920; doi:10.1371/journal.pone.0084866)
Supplement: Checklist S1 — CONSORT Checklist. (DOC) [file pone.0084866.s001.doc]

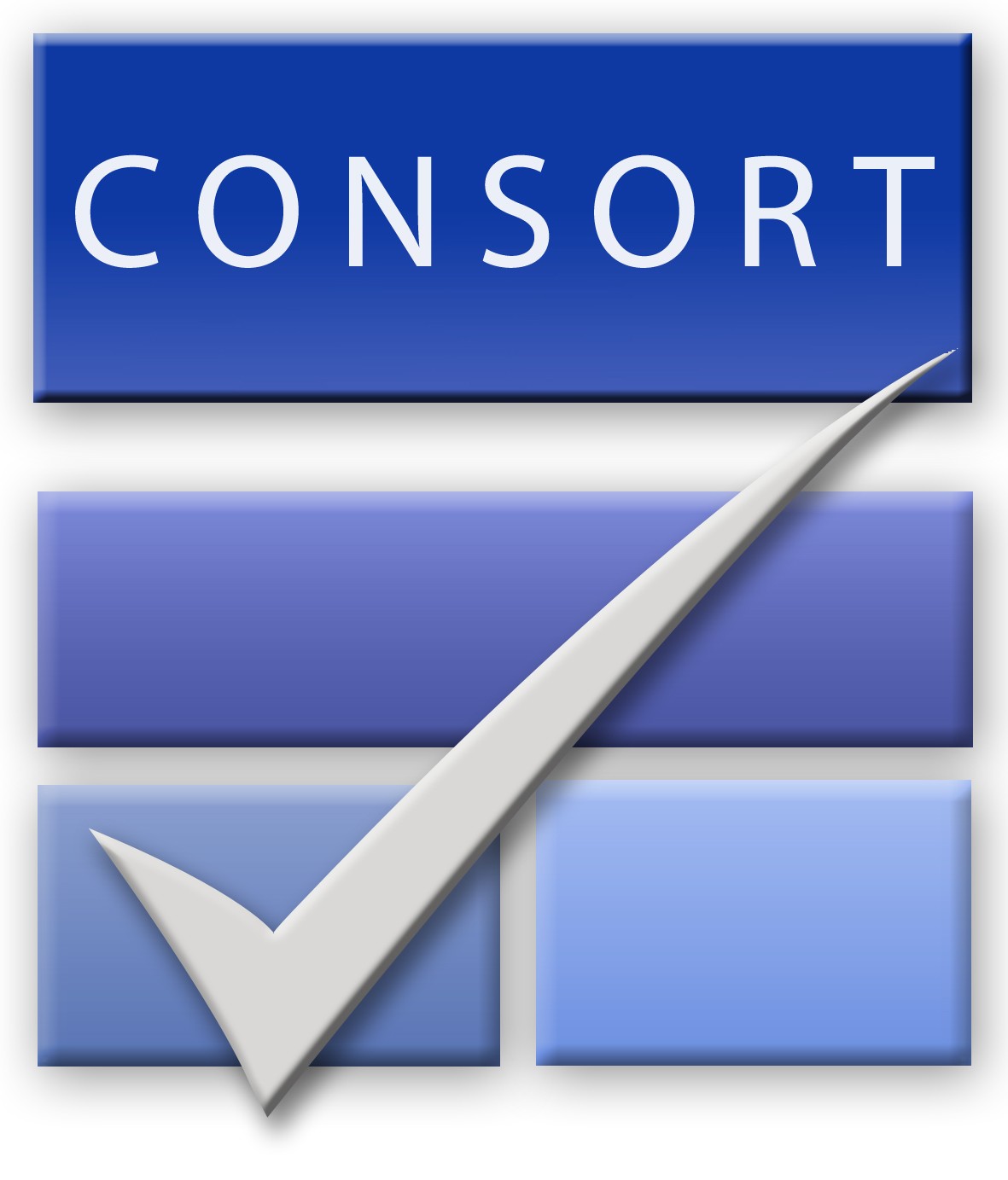
CONSORT 2010 checklist of information to include when reporting a randomised trial*

| Section/Topic | Item No | Checklist item | Reported on section |
| --- | --- | --- | --- |
| Title and abstract | | | |
|  | 1a | Identification as a randomised trial in the title | title |
|  | 1b | Structured summary of trial design, methods, results, and conclusions (for specific guidance see CONSORT for abstracts) | Abstract |
| Introduction | | | |
| Background and objectives | 2a | Scientific background and explanation of rationale | Introduction |
|  | 2b | Specific objectives or hypotheses | End of introduction |
| Methods | | | |
| Trial design | 3a | Description of trial design (such as parallel, factorial) including allocation ratio | Methods  *Study design*  *Study setting* |
|  | 3b | Important changes to methods after trial commencement (such as eligibility criteria), with reasons | N/A |
| Participants | 4a | Eligibility criteria for participants | *Patient recruitment* |
|  | 4b | Settings and locations where the data were collected | *Study setting* |
| Interventions | 5 | The interventions for each group with sufficient details to allow replication, including how and when they were actually administered | *Randomisation*  *Treatments under study*  *Sample size*  *Pharmacokinetic (PK) sampling and drug analysis* |
| Outcomes | 6a | Completely defined pre-specified primary and secondary outcome measures, including how and when they were assessed | *Pharmacokinetic analysis*  *Analysis and statistic* |
|  | 6b | Any changes to trial outcomes after the trial commenced, with reasons | N/A |
| Sample size | 7a | How sample size was determined | *Sample size* |
|  | 7b | When applicable, explanation of any interim analyses and stopping guidelines | N/A |
| Randomisation: |  |  |  |
| Sequence generation | 8a | Method used to generate the random allocation sequence | *Randomisation* |
|  | 8b | Type of randomisation; details of any restriction (such as blocking and block size) | *Randomisation* |
| Allocation concealment mechanism | 9 | Mechanism used to implement the random allocation sequence (such as sequentially numbered containers), describing any steps taken to conceal the sequence until interventions were assigned | *Randomisation* |
| Implementation | 10 | Who generated the random allocation sequence, who enrolled participants, and who assigned participants to interventions | *Randomisation* |
| Blinding | 11a | If done, who was blinded after assignment to interventions (for example, participants, care providers, those assessing outcomes) and how | N/A |
|  | 11b | If relevant, description of the similarity of interventions | N/A |
| Statistical methods | 12a | Statistical methods used to compare groups for primary and secondary outcomes | *Pharmacokinetic analysis*  *Analysis and statistic* |
|  | 12b | Methods for additional analyses, such as subgroup analyses and adjusted analyses | *Pharmacokinetic analysis*  *Analysis and statistic* |
| Results | | | |
| Participant flow (a diagram is strongly recommended) | 13a | For each group, the numbers of participants who were randomly assigned, received intended treatment, and were analysed for the primary outcome | *Patient demographic and clinical characteristics* |
|  | 13b | For each group, losses and exclusions after randomisation, together with reasons | *Patient demographic and clinical characteristics* |
| Recruitment | 14a | Dates defining the periods of recruitment and follow-up | *Patient recruitment* |
|  | 14b | Why the trial ended or was stopped | N/A |
| Baseline data | 15 | A table showing baseline demographic and clinical characteristics for each group | *Table 1 – Base-line characteristics of HIV-infected tuberculosis patients in Vietnam* |
| Numbers analysed | 16 | For each group, number of participants (denominator) included in each analysis and whether the analysis was by original assigned groups | 14-15, 28  *Rifabutin and 25-O-desacetylrifabutin pharmacokinetics*  *Lopinavir and ritonavir pharmacokinetics*  *Table 2 - Pharmacokinetic parameters of rifabutin and 25-O desacetyl rifabutin* |
| Outcomes and estimation | 17a | For each primary and secondary outcome, results for each group, and the estimated effect size and its precision (such as 95% confidence interval) | *Rifabutin and 25-O-desacetylrifabutin pharmacokinetics*  *Lopinavir and ritonavir pharmacokinetics*  *Adverse events*  *Response to treatment* |
|  | 17b | For binary outcomes, presentation of both absolute and relative effect sizes is recommended | N/A |
| Ancillary analyses | 18 | Results of any other analyses performed, including subgroup analyses and adjusted analyses, distinguishing pre-specified from exploratory | *Response to treatment* |
| Harms | 19 | All important harms or unintended effects in each group (for specific guidance see CONSORT for harms) | *Adverse events*  *Table 5: Serious adverse events in patients who completed all pharmacokinetic assessments (N=25) and in patients did not complete the assessments (N=8)* |
| Discussion | | | |
| Limitations | 20 | Trial limitations, addressing sources of potential bias, imprecision, and, if relevant, multiplicity of analyses | *Discussion* |
| Generalisability | 21 | Generalisability (external validity, applicability) of the trial findings | *Discussion* |
| Interpretation | 22 | Interpretation consistent with results, balancing benefits and harms, and considering other relevant evidence | *Discussion* |
| Other information | | |  |
| Registration | 23 | Registration number and name of trial registry | NCT00651066 |
| Protocol | 24 | Where the full trial protocol can be accessed, if available | Supporting Document |
| Funding | 25 | Sources of funding and other support (such as supply of drugs), role of funders | *Cover letter* |

*We strongly recommend reading this statement in conjunction with the CONSORT 2010 Explanation and Elaboration for important clarifications on all the items. If relevant, we also recommend reading CONSORT extensions for cluster randomised trials, non-inferiority and equivalence trials, non-pharmacological treatments, herbal interventions, and pragmatic trials. Additional extensions are forthcoming: for those and for up to date references relevant to this checklist, see [www.consort-statement.org](http://www.consort-statement.org/).
