# Supplementary material for: Randomised Pharmacokinetic Trial of Rifabutin with Lopinavir/Ritonavir-Antiretroviral Therapy in Patients with HIV-Associated Tuberculosis in Vietnam
Source: PLoS One. 2014 Jan 22;9(1):e84866. doi: 10.1371/journal.pone.0084866 (PMC3898920; doi:10.1371/journal.pone.0084866)
Supplement: Protocol S1 — Trial protocol. (PDF) [file pone.0084866.s002.pdf]

# Pharmacokinetics of Rifabutin Combined with Antiretroviral Therapy in the Treatment of Tuberculosis Patients with HIV Infection in Ho Chi Minh City, Vietnam – A phase II Trial

## ANRS 12150b

**Clinical Trial Protocol**

Version n°4.1 dated 14-02-2011

Version n°3.0 dated 09 July 2010 / 3.1 dated 25 August 2010, amendment of

Version n°2.0 dated 15 September 2008, approved:

On 14/05/2008 by Pham Ngoc Thach Hospital Ethical Committee

On 09/06/2008 by Pasteur Institute Ethical Committee

On 13/10/2008 by Vietnam Ministry of Health Ethical Committee

On 13/10/2008 by Vietnam Ministry of Health, re-approved on 21/04/2010

On 21/06/2008 by The International Union Against Tuberculosis & Lung Disease Ethical Advisory Group


**Principal Investigators**

**Pr Anthony Harries**
The International Union Against Tuberculosis and Lung Diseases (IUATLD)
68 boulevard Saint-Michel
75006 Paris, France
Tel: + 33 1 44 32 03 60
Fax : + 33 1 46 33 90 87
E-mail : adharries@theunion.org

**Dr Nguyen Huy Dung**
Pham Ngoc Thach Hospital,
120 Hung Vuong, Ward 12, District 5
Ho Chi Minh City, Vietnam
Tel : + 84 8 8550207
Fax : + 84.8 8574264
E-mail: bvpnt@vnn.vn
bshuydung@yahoo.com

**Sponsor:**

**Agence Nationale de Recherches sur le SIDA et les Hépatites Virales**
101 rue de Tolbiac
75013 Paris
Tel : +33 (0)1 53 94 60 00
Fax : +33 (0)1 53 94 60 02

**Partners:**

**Dr Truong Thi Xuan Lien**
Pasteur Institute Ho Chi Minh City
167 Pasteur Street, Ward 8, District 3,
Ho Chi Minh City
Vietnam
Tel: + 84 8 8222883,
Fax: + 84 8 8243335
E-mail: truongxuanlien@gmail.com

**Dr Anne-Marie Taburet**
Service de la Pharmacie
CHU de Bicêtre
78 rue du Gl Leclerc
94275 Kremlin-Bicêtre Cedex, France
Tel: + 33 1 45 21 29 57
Fax: + 33 1 45 21 28 60
E-mail: anne-marie.taburet@bct.ap-hop-paris.fr

**Pharmacokinetics of Rifabutin Combined with Antiretroviral Therapy in the Treatment of Tuberculosis Patients with HIV Infection in Ho Chi Minh City, Vietnam – A phase II Trial**

# ANRS 12150b

**Description of Participating Teams**

## Principal Investigators

**Pr Anthony Harries**
The International Union Against Tuberculosis and Lung Diseases (IUATLD)
68 boulevard Saint-Michel
75006 Paris, France
Tel: + 33 1 44 32 03 60
Fax: + 33 1 46 33 90 87
E-mail: adharries@theunion.org

**Dr Nguyen Huy Dung**
Pham Ngoc Thach Hospital,
120 Hung Vuong, Ward 12, District 5
Ho Chi Minh City, Vietnam
Tel: + 84 8 8550207
Fax: + 84.8 8574264
E-mail: bvpnt@vnn.vn
bshuydung@yahoo.com

## COORDINATING INVESTIGATORS:

| | |
|---|---|
| **Pr Anthony Harries**<br>The International Union Against Tuberculosis and Lung Diseases (IUATLD)<br>68 boulevard Saint-Michel<br>75006 Paris, France<br>Tel: + 33 1 44 32 03 60<br>Fax: + 33 1 46 33 90 87<br>E-mail: adharries@theunion.org | **Dr Hoang thi Quy**<br>Pham Ngoc Thach Hospital,<br>120 Hung Vuong, Ward 12, District 5<br>Ho Chi Minh City,<br>Vietnam<br>Tel: + 84 8 8550207<br>Fax: + 84 8 8574264<br>E-mail: bsquyttpnt@hcm.vnn.vn |

## SITE CLINICAL COORDINATION:

| | |
|---|---|
| **Dr Didier LAUREILLARD**<br>ANRS/Pham Ngoc Thach Hospital,<br>120 Hung Vuong, Ward 12, District 5<br>Ho Chi Minh City<br>Tel: + 84 169 7 061 034<br>Fax : +<br>E-mail: didier.laureillard@yahoo.fr | **Dr Nguyen Huu Lan**<br>Pham Ngoc Thach Hospital,<br>120 Hung Vuong, Ward 12, District 5<br>Ho Chi Minh City<br>Tel: + 84 8 8550207<br>Fax: + 84 8 8574264<br>E-mail: nguyenhuulan1965@yahoo.com.vn |

## PHARMACOLOGICAL COORDINATION:

| | |
|---|---|
| **Dr Anne-Marie Taburet**<br>Service de la Pharmacie<br>CHU de Bicêtre<br>78 rue du Gl Leclerc<br>94275 Kremlin-Bicêtre Cedex, France<br>Tel: + 33 1 45 21 29 57<br>Fax: + 33 1 45 21 28 60<br>E-mail: anne-marie.taburet@bct.ap-hop-paris.fr | **Dr Truong Thi Xuan Lien**<br>Institut Pasteur Ho Chi Minh City<br>167 Pasteur Street, Ward 8, District 3,<br>Ho Chi Minh City<br>Vietnam<br>Tel: + 84 8 8222883,<br>Fax: + 84 8 8243335<br>E-mail: truongxuanlien@gmail.com |

### METHODOLOGICAL COORDINATION:


| | |
|---|---|
| **Dominique Lagarde**<br>The International Union Against Tuberculosis and Lung Diseases (IUATLD)<br>68 boulevard Saint-Michel<br>75006 Paris, France<br>Tel: + 33 1 44 32 03 60<br>Fax: + 33 1 46 33 90 87<br>E-mail: dlagarde@theunion.org | **Dr Nguyen Thi Ngoc Lan**<br>Pham Ngoc Thach Hospital,<br>120 Hung Vuong, Ward 12, District 5<br>Ho Chi Minh City<br>Vietnam<br>Tel: + 84 8 8550207<br>Fax: + 84 8 8574264<br>E-mail: ngoclan0456@yahoo.com |


### CLINICAL BIOLOGY COORDINATION:


| | |
|---|---|
| | **Dr. Nguyen Ngoc Lan**<br>Pham Ngoc Thach Hospital,<br>120 Hung Vuong, Ward 12, District 5<br>Ho Chi Minh City, Vietnam<br>Tel: + 84 8 8550207<br>Fax: + 84 8 8574264<br>E-mail : ngoclan276@yahho.com |


### BACTERIOLOGY TEAM:


| | |
|---|---|
| | **Dr Nguyen Thi Ngoc Lan**<br>Pham Ngoc Thach Hospital,<br>120 Hung Vuong, Ward 12, District 5<br>Ho Chi Minh City<br>Vietnam<br>Tel: + 84 8 8550207<br>Fax: + 84 8 8574264<br>E-mail: ngoclan0456@yahoo.com |


### CLINICAL TEAM:


| | |
|---|---|
| | **Nguyen Huu Minh (Clinician)**<br>Recruitment, Clinical follow-up and monitoring<br>Pham Ngoc Thach Hospital,<br>Tel: + 84 8 8550207<br>Fax: + 84 8 8574264<br><br>**Nguyen Thi Oanh (Trial Nurse)**<br>Trial Nurse and data entry.<br>Pham Ngoc Thach Hospital<br><br>**Dr To My Huong (Data Monitoring)**<br>Data Verification<br>Pham Ngoc Thach Hospital |

### ADMINISTRATIVE COORDINATION:



| | |
|---|---|
| **Dominique Lagarde**<br>The International Union Against Tuberculosis and Lung Diseases (IUATLD)<br>68 boulevard Saint-Michel<br>75006 Paris, France<br>Tel: + 33 1 44 32 03 60<br>Fax: + 33 1 46 33 90 87<br>E-mail : dlagarde@theunion.org | **Dr Nguyen Thi Ngoc Lan**<br>Pham Ngoc Thach Hospital,<br>120 Hung Vuong, Ward 12, District 5<br>Ho Chi Minh City<br>Vietnam<br>Tel: + 84 8 8550207<br>Fax: + 84 8 8574264<br>E-mail: ngoclan0456@yahoo.com |



### INVESTIGATORS:



| | |
|---|---|
| **Dr Nguyen Hong Duc**<br>Pham Ngoc Thach Hospital,<br>120 Hung Vuong, Ward 12, District 5<br>Ho Chi Minh City, Vietnam<br>Tel: + 84 8 8550207<br>Fax: + 84 8 8574264<br>E-mail: bsquyttpnt@hcm.vnn.vn | **Dr Nguyen Thi Nguyet Thu**<br>Institut Pasteur Ho Chi Minh City<br>167 Pasteur Street, Ward 8, District 3,<br>Ho Chi Minh City, Vietnam<br>Tel: + 84 8 8222883,<br>Fax: + 84 8 8243335<br>E-mail: ntnguyetthu05@yahoo.com |



### SPONSOR REPRESENTATIVE:



| | |
|---|---|
| **Dr Claire Rekacewicz**<br>ANRS<br>101 rue de Tolbiac<br>75013 Paris, France<br>Tel:<br>Fax:<br>E-mail: | **Titre, Nom**<br>ANRS<br>101 rue de Tolbiac<br>75013 Paris, France<br>Tel:<br>Fax:<br>E-mail: |



### PARTNERS:



| | |
|---|---|
| **Dr Alexander Pym**<br>Medical Research Council<br>Unit for Clinical and Biomedical TB Research, 491 Ridge Road, PO Box 70380<br>Overport, Durban, 4067, South Africa<br>Tel : +27 31 2034745<br>Fax : + 27 31 2034702<br>E-mail : Alexander.Pym@mrc.ac.za | |

**Pharmacokinetics of Rifabutin Combined with Antiretroviral Therapy in the Treatment of Tuberculosis Patients with HIV Infection in Ho Chi Minh City, Vietnam – A phase II trial**

# ANRS 12150

## GLOSSARY

| | |
|---|---|
| AE | Adverse Events |
| AFB | Acid Fast Bacilli |
| AIDS | Acquired Immuno Deficiency Syndrome |
| ARR | Acquired Rifamycin Resistance |
| ART | Anti Retroviral Therapy |
| ARVs | Anti retrovirals |
| AUC | Area under the curve |
| BD | Twice a day |
| $C_{max}$ | Maximum Concentration |
| CRF | Case report form |
| CTD | Clinical Trial Division |
| CXR | Chest x-ray |
| DMID | Division of Microbiology and Infectious Diseases |
| DSMC | Data Safety and Monitoring Committee |
| DST | Drugs Sensitivity Testing |
| E, EMB | Ethambutol |
| EFV | Efavirenz |
| FBC | Full blood count |
| GCP | Good clinical practice |
| H, INH | Isoniazid |
| HIV | Human immunodeficiency virus |
| IRIS | Immune Reconstitution Inflammatory Syndrome |
| IUATLD | International Union Against Tuberculosis and Lung Diseases |
| LFT | Liver Function Tests |
| LPV | Lopinavir |
| LPV/r | Lopinavir/ritonavir |
| MDR TB | Multidrug resistant tuberculosis |
| MRC | Medical Research Council |
| NNRTI | Non Nucleoside Reverse Transcriptase Inhibitor |

| | |
|---|---|
| NRTI | Nucleoside Reverse Transcriptase Inhibitor |
| NVP | Nevirapine |
| OD | Once-a-day |
| OI | Opportunistic infection |
| PK | Pharmacokinetic |
| PZA | Pyrazinamide |
| RBT | Rifabutin |
| RMP | Rifampicin, Rifampin |
| SAE | Serious Adverse Event |
| SCC | Short course chemotherapy |
| TB | Tuberculosis |
| TDR | Tropical disease research |
| VCT | Voluntary Counselling and Testing |
| VL | Viral load |
| WHO | World Health Organisation |

**Etude Pharmacocinétique de la Rifabutine combinée avec la Thérapie Antirétrovirale dans le Traitement de Patients atteints de Tuberculose avec co-infection par le VIH à Ho Chi Minh City, Vietnam: essai en Phase II**

## ANRS 12150

### Résumé détaillé

Objectifs :

Principal : déterminer la pharmacocinétique de la Rifabutine (RBT) en combinaison avec les médicaments anti-rétroviraux (ARV) chez le patient tuberculeux infecté par le VIH à un stade avancé au Vietnam.

Secondaires : 1. Comparer la biodisponibilité de deux doses différentes de RBT en combinaison avec Lopinavir "boosté" (LPV/r), avec la biodisponibilité de RBT sans ARV;
2. Déterminer la biodisponibilité de LPV/r en combinaison avec RBT;
3. Déterminer l'efficacité du traitement de la tuberculose
4. Déterminer la sécurité et la toxicité de la RBT combinée aux ARV durant la phase initiale du traitement de la tuberculose (TB).
5. Evaluer les différences génétiques pouvant influencer les réponses aux traitements antirétroviraux et antituberculeux

Méthodologie :
Essai clinique randomisé, multi-doses, *cross-over*, à 2 bras.

Critères d'inclusion : patients avec TB pulmonaire nouvellement diagnostiquée et sérologie VIH positive,

Les patients seront éligibles pour l'inclusion dans l'essai s'ils remplissent les conditions suivantes:

i. Un diagnostic de TB pulmonaire défini comme: a) Au moins 2 frottis de crachats positifs (égal ou supérieur au grade 1 pour le nombre de BAAR; ou b) 1 frottis de crachat BAAR positif et une radiographie du thorax compatible avec une TB active ; ou c) une culture de crachats positive pour *M. tuberculosis* et une radio compatible avec une TB active; ou d) 1 radiographie du thorax compatible avec une tuberculose active et des antécédents cliniques compatibles avec une tuberculose et 2 frottis de crachats BAAR négatifs
ii. Avoir un test de sérologie du VIH positif, et un taux de CD4 lymphocyte inférieur ou égal à $250/mm^3$ lors de la visite de pré-screening ;
iii. Poids > 40 Kg.
iv. Ne jamais avoir pris de traitement ARV;
v. Ne pas présenter d'anomalie clinique ou de laboratoire de grade 3 ou 4 (selon le tableau de classification DMID) contre-indiquant la participation à l'essai;
vi. Pour les femmes en âge de procréer, avoir un test de grossesse négatif le jour du recrutement et accepter de prendre les mesures contraceptives appropriées pendant la durée de l'essai ;
vii. Avoir une adresse définie, avec un accès aisé ;
viii. Avoir un score de Karnofsky ≥ 80%

Critères de non-inclusion :

Les patients qui présentent l'une quelconque des conditions suivantes ne seront **pas** éligibles pour l'inclusion dans l'essai:

i. Antécédent de TB dans l'année précédente ou de traitement de TB multi-résistante à quelque moment que cela soit
ii. Une infection opportuniste quelle qu'elle soit, demandant un traitement anti-infectieux approprié
iii. une ou des contre-indications pour l'un quelconque des médicaments de l'essai;
iv. Un diabète demandant un traitement spécifique
v. Une toxicomanie (drogue ou alcool) risquant d'affecter l'adhérence au traitement
vi. Des antécédents d'hypersensibilité à l'un quelconque des médicaments
vii. Une maladie mentale qui diminuerait la capacité de donner un consentement éclairé ou résulterait en une mauvaise observance du protocole ou du traitement
viii. Une neutropénie (< 1200 cellules/L)
ix. Anémie (hémoglobine <6.8 g/dl)
x. Une condition requérant un ou des médicaments qui pourraient interagir avec les médicaments de l'essai
xi. des tests de fonction hépatique > grade 2 des tableaux DMID,
xii. une grossesse ou un allaitement
xiii. un score de Karnofsky score <80%
xiv. toute condition rendant le malade incapable de comprendre la nature, les objectifs, ou les conséquences possible de cette étude ou de donner son consentement.

Traitements et schéma de l'essai :

Les patients reçoivent la Rifabutine, 300mg 1 fois par jour, à la place de la Rifampicine durant les 2 premières semaines de la phase intensive du traitement tuberculeux. Après ces deux semaines, la $1^{ère}$ étude pharmacocinétique (PK1) sera réalisée. Les patients seront alors randomisés pour recevoir une combinaison de RBT et d'ARV, avec des doses différentes de RBT:

- A: NRTI + LPV/r (2 cp x 2/j) + RBT (150 mg x 3/sem);
- B: NRTI + LPV/r (2 cp x 2/j) + RBT (150 mg x 1/j)

La moitié des patients recevra le traitement (A), puis sera changé pour le traitement (B) après trois semaines, l'autre moitié recevant l'inverse (B puis A). La $2^{ème}$ étude pharmacocinétique (PK2) sera réalisée trois semaines après PK1, et la $3^{ème}$ (PK3), trois semaines après PK2, c'est-à-dire à la fin de la phase intensive.

Après la PK3, pour toute la phase d'entretien du traitement de la tuberculose, les patients sont référés au Programme National de la Tuberculose et au Programme National VIH afin d'être traités selon les recommandations. La rifabutine sera changée au profit de la rifampicine et le lopinavir/r par de l'efavirenz. Ainsi les patients seront traités selon les recommandations du Programme National Tuberculose et du Programme National SIDA et suivis dans ce cadre. Une visite de fin d'étude est prévue à la fin du traitement de la tuberculose, à entre S24 et S26.

Critères de jugement : La biodisponibilité de la RBT sera mesurée par l'$AUC_{0-24}$ et comparée à l'$AUC_{0-24}$ pour chaque dose de RBT en combinaison avec l'ARV. De plus, la biodisponibilité du Lopinavir combiné à 2 doses de RBT sera déterminée par l'$AUC_{0-12}$ .

Nombre de sujets nécessaire : 32 patients

Durée de l'essai :

temps de participation par patient : 6 mois
durée des inclusions : 6 mois
durée totale de l'essai : 15 mois

<u>Dates prévisionnelles</u> :

| | |
|---|---|
| Soumission aux autorités pour approbation : | 15 avril 2008 |
| Approbation complète reçue : | 23 mars 2009 |
| Approbation du Ministère de la Santé Vietnamien | 13 octobre 2008 |
| Re-approbation du Ministère de la Santé Vietnamien | 21 avril 2010 |
| Début du recrutement: | 25 juin 2010 |
| Interruption de l'enrôlement: | 19 octobre 2010 |
| Soumission amendement version 4.2: | 20 avril 2011 |
| Approbation du Ministère de la santé: | 15 juin 2011 |
| Reprise des inclusions: | 15 août 2011 |
| Fin des inclusions: | 15 février 2012 |
| Fin du Suivi: | 15 août 2012 |
| Fin de l'analyse: | 15 novembre 2012 |

+ Deux mois additionnels en fonction des contingences

**Pharmacokinetics of Rifabutin Combined with Antiretroviral Therapy in the Treatment of Tuberculosis Patients with HIV Infection in Ho Chi Minh City, Vietnam: a phase II trial**

## ANRS 12150

**Detailed Summary**

Objectives:

Primary: to determine the pharmacokinetics of RBT in combination with ARVs in Vietnamese HIV infected patients with pulmonary TB.

Secondary:
1. to compare the bioavailability of two different doses of RBT in combination with LPV/r, with the bioavailability of RBT taken without ARVs;
2. to determine the bioavailability of LPV/r in combination with RBT;
3. to determine the outcomes of tuberculosis treatment
4. to determine the safety and toxicity of RBT in combination with ARV during the initial phase of tuberculosis treatment.
5. to assess the genetic differences that may affect the response to antiretroviral and tuberculosis treatments.

Methodology:
Randomized, open-label, multi-dose, 2-arm, cross-over clinical trial

Inclusion criteria: newly-diagnosed TB patients with HIV infection

Patients will be eligible for inclusion in the trial if they fulfil the following criteria:

i. Having a diagnosis of pulmonary tuberculosis defined as: a) At least 2 sputum smears positive (equal to or exceeding grade 1) for AFB; or b) 1 sputum smear positive for AFB and a chest radiograph (CXR) compatible with active tuberculosis: or c) 1 sputum culture positive for *M. tuberculosis* and a CXR compatible with active tuberculosis: or d) a CXR compatible with active tuberculosis and a clinical history compatible with tuberculosis and 2 sputums smear negative for AFB.
ii. Positive HIV antibody test and a CD4 lymphocyte count less than or equal to 250/mm$^3$ at the pre-screening visit.
iii. Weight > 40 Kg.
iv. Having had no ART at all
v. Having no grade 3 or 4 clinical or laboratory findings according to DMID tables.
vi. If a female of childbearing age, having a negative pregnancy test on day of enrolment and willing to take appropriate contraceptive measures during the duration of the trial.
vii. Having a firm home address that is readily accessible
viii. Have a Karnofsky score Q≥ 80%

Non inclusion criteria:

Patients who meet any of the following criteria will not be eligible for the study:

i. History of TB (within previous year) or MDR TB treatment (at anytime)
ii. Concomitant opportunistic infection requiring additional anti-infectious treatment
iii. A formal contraindication to any drug used in the trial
iv. Diabetes mellitus requiring drug treatment
v. Recreational drug or alcohol abuse that is likely to affect drug adherence
vi. History of drug hypersensitivity to TB or related medications
vii. Mental illness that could impair ability to give informed consent or result in poor adherence to trial protocol and therapy.
viii. Neutropenia (total neutrophil count < 1200 cells/L)
ix. Anaemia (haemoglobin <6.8 g/dL)
x. Requiring concomitant medications that may potentially interact with study drugs
xi. Liver Function Test > grade 2 DMID tables,
xii. Pregnant or lactating women
xiii. Karnofsky score Q<80%
xiv. Any condition rendering the patient unable to understand the nature, scope, and possible consequences of this study and to provide consent.

Treatments of the Trial:

Patients will have Rifampin replaced by Rifabutin 300mg once a day (OD) during the first two weeks of intensive phase of tuberculosis treatment. After two weeks, the first PK study (PK1) will be completed. Patients will then be randomized to receive a combination of RBT and ARV, with different doses of RBT:

- A: NRTI + LPV/r (2 tabs twice a day) + RBT (150 mg TPW);
- B: NRTI + LPV/r (2 tabs twice a day) + RBT (150 mg OD)

Half the patients will be allocated to sub-treatment (A) and then switched to treatment (B) after three weeks, and half will be allocated the reverse (B then A). PK2 takes place three weeks after PK1 and PK3 three weeks after PK2, at the end of the intensive phase

After the PK3, during the continuous phase of tuberculosis treatment, participants will be referred to National Tuberculosis Program and to National AIDS Program to be treated according to standard care. Rifabutin will be switched to rifampicin and lopinavir/rtv to efavirenz. Thus patients will be treated and followed according to recommendations from and by the National TB Program and by the National AIDS program. An end study visit will be done at the end of the TB treatment between W24 and W26.

End-points: The bioavailability of RBT will be measured by the $AUC_{0-24}$ and compared to the $AUC_{0-24}$ for each of the RBT doses in combination with the ARV. In addition, the bioavailability of the LPV in combination with two doses of RBT will be determined by the $AUC_{0-12}$.

Sample size: 32 patients

Duration of the trial:

Average time per patient: 6 months
Duration of inclusion: 6 months
Total duration of trial: 15 months

Provisional dates:

| | |
|---|---|
| Submission to authorities: | 15 April 2008 |
| Full approval received: | 23 March 2009 |
| Approval Vietnamese Ministry of Health: | 13 October 2008 |
| Re-approval Vietnamese Ministry of Health: | 21 April 2010 |
| Start recruitment: | 25 June 2010 |
| Enrolment interruption: | 19 October 2010 |
| Submission protocol version 4.2: | 20 April 2011 |
| Expected approval by Vietnamese Ministry of Health: | 15 June 2011 |
| Start enrolment: | 15 August 2011 |
| Finish enrolment: | 15 February 2012 |
| Complete follow-up: | 15 August 2012 |
| Complete analysis and reporting: | 15 November 2012 |

+ Additional 2 months contingency

**Pharmacokinetics of Rifabutin Combined with Antiretroviral Therapy in the Treatment of Tuberculosis Patients with HIV Infection in Ho Chi Minh City, Vietnam – A phase II trial**

# ANRS 12150

## TABLE OF CONTENTS

**DESCRIPTION OF PARTICIPATING TEAMS** ..... **2**
**GLOSSARY** ..... **5**
**RESUME DETAILLE** ..... **7**
**DETAILED SUMMARY** ..... **10**
**TABLE OF CONTENTS** ..... **13**
**I. BACKGROUND AND RATIONALE:** ..... **14**
**II. PROJECT OBJECTIVES:** ..... **19**
**2.1 PRIMARY OBJECTIVE:** ..... **19**
**2.2 SECONDARY OBJECTIVES:** ..... **19**
**III. STUDY DESIGN** ..... **19**
**1. SELECTION OF PATIENTS:** ..... **20**
1.1 PRE-SCREENING ..... 20
1.2 SCREENING ..... 20
1.3 INCLUSION: ..... 20
*1.3.1. Inclusion Criteria:* ..... *20*
*1.3.2. Non inclusion Criteria:* ..... *21*
1.4 RANDOMISATION ..... 21
**2. TREATMENTS UNDER STUDY** ..... **22**
2.1 TREATMENT ARMS ..... 22
2.2. ARV SELECTION: ..... 24
2.3 RBT DOSING ..... 24
**3. OUTCOME MEASURES** ..... **25**
3.1 PRIMARY OUTCOMES: ..... 25
3.2 SECONDARY OUTCOMES: ..... 25
**4. SAMPLE SIZE** ..... **25**
**5. INVESTIGATIONAL PLAN:** ..... **25**
5.2 TRIAL SCHEDULE ..... 26
5.3 RECRUITMENT PROCEDURES: ..... 28
5.4 FOLLOW-UP PROCEDURES: ..... 28
5.5 ADVERSE EVENTS (AEs) AND SEVERE ADVERSE EVENTS (SAEs): ..... 29
5.6 MICROBIOLOGY PROCEDURES: ..... 30
5.7 IMMUNO-VIROLOGICAL PROCEDURES: ..... 30
5.8 PHARMACOLOGICAL PROCEDURES AND ASSAYS: ..... 30
5.9 PHARMACOKINETIC ANALYSIS: ..... 31
5. 10 PHARMACOGENOMICS SAMPLES ..... 32
5.11 STORAGE OF SAMPLES ..... 32
**6. TRIAL MANAGEMENT:** ..... **33**
**7. ETHICAL ASPECTS:** ..... **34**
**8. PROCUREMENT OF DRUGS AND STUDY MATERIAL:** ..... **34**
**9. TRIAL SITE AND COLLABORATING INSTITUTIONS:** ..... **35**
9.1 OUTPATIENT DEPARTMENT, ..... 35
9.2 DEPARTMENT OF BIOLOGICAL ANALYSIS, PASTEUR ..... 35
9.3 DEPARTMENT OF PHARMACOLOGY, HOSPITAL KREMLIN BICÊTRE, PARIS: ..... 35
9.4 INTERNATIONAL UNION AGAINST TUBERCULOSIS AND LUNG DISEASE, PARIS, FRANCE ..... 36
**10. PUBLICATION POLICY** ..... **36**
**APPENDIX 1** ..... **39**
2. DEPARTMENT OF BIOLOGICAL ANALYSIS, PASTEUR INSTITUTE, HO CHI MINH CITY: ..... 40
3 DEPARTMENTS OF PHARMACOLOGY, HOPITAL KREMLIN BICÊTRE, PARIS: ..... 41
4. INTERNATIONAL UNION AGAINST TUBERCULOSIS AND LUNG DISEASE, PARIS, FRANCE ..... 42
**APPENDIX II:** ..... **43**
**SERIOUS ADVERSE EVENTS REPORTING PROCEDURE FOR ANRS SPONSORED RESEARCH** ..... **43**
**IN DEVELOPING COUNTRIES** ..... **43**

## I. Background and rationale:

In countries having a high incidence of active tuberculosis (TB) and localized or generalized HIV epidemic, TB is the one of the most serious opportunistic infections (OI) and the leading cause of death among HIV-infected patients (1). The HIV epidemic and the resulting increase in HIV-associated TB cases is now challenging or even reversing NTP achievements in TB control in the countries worst affected by these overlapping epidemics (2). Viet Nam is ranked 13th among the WHO list of 22 high-burden countries, based upon estimated total number of tuberculosis cases (3) and 37% of admitted HIV infected patients presented with tuberculosis (4). The prevalence of HIV infection in general population is 0,2 – 0,8% and the epidemic is of the concentrated type affecting mostly intravenous drug users, prostitutes, their clients and partners of the clients (5).  HIV prevalence in adult TB cases increased steeply from 0.5% in 1995 to 4% in 2000 and reach 14% in certain locations (6). In view of the frequent TB/HIV co-infection, there is a clear need to address treatment of both diseases in the same individual.

### Combining ART and TB chemotherapy

Short Course Chemotherapy (SCC) for tuberculosis usually involves two months (intensive phase) of rifampin (RMP), isoniazid (H), ethambutol (E) and pyrazinamide (Z), followed by 4 months (continuation phase) of RMP and H. Current WHO guidelines recommend to start ART in HIV infected tuberculosis patients within 2 to 8 weeks of SCC in all TB patients irrespective of the CD4 cell count. Combining these drugs is, however, fraught with many difficulties including drug interactions, cumulative toxicities, pill burden and paradoxical reactions, such as the Immune Reconstitution Inflammatory Syndrome (IRIS) (*7, 8*). Devising strategies to overcome these difficulties is a clinical research priority for countries with high burdens of TB and HIV.

The most important drug interactions arise because RMP is a potent inducer of the hepatic cytochrome CYP450 enzyme system that reduces the plasma concentration of co-administered drugs metabolized through this pathway. This includes the NNRTIs, nevirapine (NVP) and efavirenz (EFV) as well as PIs such as boosted lopinavir (LPV/r) (*9*). RMP reduces EFV bioavailability by approximately 25%, but this can be countered by increasing the daily dose from 600mg to 800mg (*7*), although in some populations the lower dose may result in adequate virological responses (*10*). However RMP has a greater effect on the bioavailability of NVP and protease inhibitors and is more problematic to correct with dose adjustments (*11, 12*). For these reasons, the interactions between SCC and ART medications could reduce adherence to and efficacy of both HIV and TB treatments. It is therefore important to determine prospectively the tolerability of different SCC/ART combinations. Given the potentially detrimental effects of initiating ART early in SCC, it was unclear what the optimal combination and timing of ART should be to maximize TB and HIV

treatment outcomes. A pivotal study (Camelia Study) to address the timing of ART in SCC has recently reported from Cambodia that the mortality of HIV infected tuberculosis patients is significantly reduced if ART are started after two weeks of SCC rather than delaying for two months, but there is also a need to determine the optimum combination of SSC+ART.

**Rationale for new SCC/ART combinations**

The current recommended standard of care for the concomitant treatment of TB/HIV is a RMP based SCC in combination with EFV and two nucleosides. A dose adjustment of EFV from 600 mg to 800 mg is recommended to compensate for the increased hepatic metabolism caused by rifampin induced enzyme induction. There are however situations in which an alternative to EFV is required:

1. EFV is poorly tolerated in some individuals because of neuropsychiatric and other side effects.
2. Some AIDS National Programmes are advocating NVP instead of EFV as first line ARV therapy for economic reasons.
3. EFV is contraindicated in women at risk of pregnancy because of uncertainties about potential teratogenicity, stemming from animal studies.
4. EFV may be sub-optimal in women who have received NVP monotherapy to prevent vertical transmission of HIV. Single point mutations in reverse transcriptase conveying cross-resistance to EFV can diminish responses to subsequent NNRTI based ART (*13*).
5. As ART roll out programmes progress, the numbers of patients failing EFV based ART will grow (*14*). Virological failure in patients on NNRTI regimens is usually associated with the viral acquisition of mutations conferring resistance to NVP and EFV. These patients will need to be treated with a PI and will also have immunological failure and increased susceptibility to tuberculosis.

The increasing number of patients failing NNRTI based therapy will lead to substantial demand for ART/SCC combinations involving a PI. The bioavailability of PIs is substantially reduced by RMP, in the case of boosted lopinavir (LPV/r) by 75 to 99%. This could be corrected by super boosting LPV with extra ritonavir but would result in high pill burden and concerns about tolerability and hepatotoxicity (*12*). Concerns also remain over hepatotoxicity and dose escalation to correct the 35% reduction of NVP bioavailability when co-administered with RMP. It is not yet clear whether the use of standard doses of NVP with RMP can produce adequate antiviral response in all populations, but this approach is in conflict with data associating ARV drug levels with virological failure. Therefore, an alternative strategy to overcome the adverse drug interactions of combinations of SSC/ART is to replace RMP with rifabutin.

**Rifabutin (RBT)**

Rifamycins are indispensable for SCC because of their unique sterilizing capacity that allowed shortening TB therapy to 6 months. Regimens without RMP, or a structurally similar rifamycin, require at least one year of therapy. All rifamycins are inducers of the hepatic cytochrome CYP450 enzyme system, but the effects of RBT are the least pronounced. RBT has a half-life approximately ten-fold longer than RMP. Its bioavailability is less than RMP, but it is less protein bound and more lipid soluble than RMP, so is more extensively distributed in tissues. Published randomized trials have found that RMP and RBT are statistically indistinguishable in terms of clinical outcomes for TB, although one study reported significantly more rapid clearance of acid-fast bacilli from sputum after two months of treatment with RBT (*15*).

The pharmacokinetic and other properties of RBT make it an attractive substitute for RMP in SCC/ART combinations and it has become the standard of care in Europe and the USA for HIV/TB patients needing a PI. Previous studies have suggested that the level of induction of CYP450 enzymes is not sufficient to require dose alteration of co-administered ARV, although studies need to be repeated to establish if this also applies in Asian populations with advanced immunodeficiency. There is also uncertainty concerning the effects of ARV on RBT, with PIs and EFV having opposite effects on RBT bioavailability, increasing and decreasing it respectively. NVP is thought to be neutral with regard to RBT bioavailability. A recent study from the University of Cape Town has also shown that HIV infection reduces RMP drug concentrations by 39% (*16*), and this effect probably applies for other rifamycins. RBT bioavailability, particularly in intermittent regimens, has been associated with the emergence of acquired rifamycin resistance in patients with low CD4 lymphocyte counts. It is therefore prudent to conduct further pharmacokinetic studies in patients with advanced immunodeficiency before proceeding to Phase III evaluation of RBT.

It is surprising that RBT has been overlooked as a key drug to counter the devastating HIV/TB epidemic in most affected countries. This is usually based on the erroneous assumption that it will be too expensive. RBT was patented for use in TB in 1981, so is eligible for generic production. Furthermore, as outlined above, the increase in bioavailability of RBT when co-administered with PIs means that the dose reductions of RBT will result in cost savings. Also, if RBT can be used with NV without concerns about adverse drug interactions, this could represent a considerable cost saving with regard to RMP which international guidelines recommend should only be used with the more expensive EFV.

The overall aim of this project is to evaluate rifabutin, as a replacement for rifampicin, in combined SCC/ART regimens. The Unit for Tuberculosis Research at the MRC and the IUATLD have already identified a consortium of partners to conduct a Phase III trial to determine the tolerability and efficacy of

RBT in SSC/ART regimens. However, *a critical first step before proceeding to phase III evaluation is to define the pharmacokinetic interactions of rifabutin with antiretrovirals.* This will ensure the optimal dose selection for a large-scale phase III trial. The aim of the present project is therefore to establish the pharmacokinetics of RBT in combination with LPV/r, in order to subsequently proceed to a phase III trial comparing the safety, tolerability and efficacy of RBT in HIV/TB infected patients.

**Pharmacogenomics**

There is considerable inter-individual variability in the disposition of drugs, and at least some is caused by polymorphisms in genes coding for drug-metabolizing enzyme and transporters. Some recent reports confirmed the need of pharmacogenetic studies to elucidate the ethnic or racial differences in drug disposition (pharmacokinetics - PK) which can occur at any step of drug disposition, absorption, distribution, transport, metabolism or excretion, drug-drug interactions and drug effect or tolerance, and pharmacodynamics (Haas, 2005, Telenti and Zanger, 2008). Any increase or decrease in drug concentrations and exposure will impact on efficacy and tolerance. The present pharmacogenetic sub-study could be helpful for a better understanding of variability in rifabutin or antiretroviral PK and of occurrence of adverse events in co infected tuberculosis and HIV Vietnamese patients included in the ANRS12150b study who accept to participate in this sub-study.

The objective of the present study is to identify in the included population single base mutations (Single Nucleotide Polymorphism SNPs) within genes involved in drug metabolism and transport. This will allow identification of patients carrying allelic variants encoding for loss of function enzymes or transporters. The SNPs will be selected according to the literature and the SNP database. The frequency of SNPs varies between populations.

The current study aim is to evaluate the best rifabutin dosing regimen when coadministered with lopinavir/ritonavir in HIV-infected patients. Lopinavir and ritonavir are CYP3A4 substrates and ritonavir is a potent CYP3A inhibitor which increases the concentrations of lopinavir.  Pharmacogenetics of antiretroviral drugs has been recently reviewed by Telenti and Zanger (2008). Unlike rifampin, some rifabutin and most of its partially active 25-desacetyl-rifabutin metabolite (hereafter, *desacetyl-rifabutin*) are cleared by CYP3A4 (Iatsimirskaia et al. 1997). Although CYP3A4 is the major form in adult liver, in some populations CYP3A5 can make a significant contribution in subjects with low CYP3A4 activity. Although polymorphism in CYP3A4 have controverstial effects on expression, CYP3A5 is highly polymorphic. The *CYP3A5*3* allele (G at position 6986) creates a cryptic splice site creating aberrant mRNA, with a premature stop codon. Individuals with at least one A allele (*CYP3A5*1*) produce high levels of full-length CYP3A5 mRNA and express an active CYP3A5 enzyme, while those carrying the *CYP3A5 6986 GG (CYP3A*3)* genotype have very low or even undetectable hepatic CYP3A5 protein content. Most Caucasian expressed the loss of function *CYP3A5 6986GG* genotype associated with a small amount of translated CYP3A5 protein with a G

allele frequency ranging from 0.87 to 0.94 in various Caucasian populations. In contrast, in various Asian populations G allele frequencies were lower ranging from 0.59 in Indians to 0.67 in Vietnamese and 0.74-0.78 in Japanese, Chinese and Koreans (Veiga et al., 2009, Hiratsuka et al. 2002). The frequency is even lower in patients of African descent (0.36) (Haas et al., 2004). Higher expression of CYP3A5 protein will lead to an increase in clearance of CYP3A substrate drugs such as HIV-1 protease inhibitors. Lower saquinavir, atazanavir or indinavir concentrations were demonstrated in patients who express CYP3A5, although disposition of lopinavir combined with ritonavir, which inhibits both CYP3A4 and CYP3A5, remains unaffected (Estrela et al., 2009). Effect of CYP3A5 genetic polymorphism on rifabutin and its metabolite disposition is currently unknown.

More recently, the effect of uptake and efflux transporters on drug disposition was recognized. P-glycoprotein (ABCB1) transporter, which is encoded by the ABCB1 (MDR 1) gene, has a broad substrate specificity and plays an important role in reducing drug bioavailability and intracellular drug concentration. A number of studies have investigated the functional consequences of variation in this gene particularly the ABCB1-3435C>T polymorphism. This variant has been associated with mRNA, protein and serum levels, and with responses to a number of drugs (Loeuillet et al., 2007). Plasma concentrations of the protease inhibitors and CD4 cell count response have been associated with the T allele at the MDR1 C3435T locus (Saitoh et al., 2005). However, other studies (Ma et al., 2007, Estrela et al., 2009) did not confirm a correlation between trough plasma concentrations of atazanavir or lopinavir and the variant T allele. More recently, SLCO1B1 (OATP, organic anion transporting polypeptide) polymorphism was found to affect lopinavir pharmacokinetics (Lubomirov et al., 2010).

Two major families of hepatic enzymes are involved in the hepatic elimination of isoniazid (N-acetyltransferase 2 (NAT-2) and Glutathion-S-Tranferases (Mu, théta et pi: GSTM1, GSTT1 et GSTP1). These are subject to marked pharmacogenetic variation, and different combinations of SNPs in the NAT and GST genes lead to different phenotypes (NAT2: low or fast acetylor). Genetic polymorphism could confer changes in liver susceptibility to those treatment combinations.

Rifamycins (rifampicin and rifabutin), are well known for their ability to induce drug-metabolizing enzymes and transporters, although rifabutin to a lesser extent, through activation of a nuclear receptor: the Pregnane X Receptor (PXR). It is now accepted that drug-dependent induction of genes important for drug disposition is mediated by nuclear receptors pregnane X receptor (PXR) (Tirona et al. 2005, which function as key ligand-activated transcription factors that control the expression of drug metabolizing enzymes and transporters. In comparison with other genes, relatively few single nucleotide polymorphisms (SNPs) are observed in hPXR. The single amino acid variants identified in subjects of Caucasian and African origin include amino acid changes in or close to the Ligand Binding Domain (R122Q (PXR*4), V140M, D163G, and A370T) and others in the N-terminal region (E18K, P27S (PXR*2), and G36R (PXR*3). The most frequent of these,

P27S, was identified in 14.9% of chromosomes from subjects of African origin, whereas the others had allele frequencies below 3%.

Our main goal is to select some “target” functional SNPs and genotype our population to identify the impact of those different genes on PK and PD variabilities. We will focus on the association between rifabutin-lopinavir/ritonavir pharmacokinetics and genetic polymorphism of CYP3A5, ABCB1, OATP1B1 and PXR genetic polymorphisms. NAT2 and GST polymorphism will be related to liver toxicity if any. Any new data on these drug pharmacogenetics or unexpected results of the pharmacokinetic/pharmacodynamic study or tolerance could lead to study other allelic variant of drug metabolizing enzymes or transporters.

## II. Project Objectives:

### 2.1 Primary objective:

To determine the pharmacokinetics of RBT in combination with ARVs in immuno-compromised patients with tuberculosis in Vietnam.

### 2.2 Secondary objectives:

1. To compare the bioavailability of two different doses of RBT in combination with LPV/r, with the bioavailability of RBT taken without ART in Vietnamese HIV infected patients with pulmonary tuberculosis and advanced immunodeficiency.

2. To determine the bioavailability of LPV/r in combination with RBT in Vietnamese TB patients with advanced immunodeficiency.

3. To determine the outcomes of tuberculosis treatment with rifabutin 150 mg once per day or thrice per week in combination with LPV/r during the initial phase of tuberculosis treatment.

4. To determine the safety and toxicity of RBT in combination with ART during the initial phase of tuberculosis therapy in Vietnamese patients.

5. To assess the genetic differences that may affect the response to antiretroviral and tuberculosis treatments.

## III. Study Design

This is a randomized, open-label, multi-dose, 2-arm, cross-over, clinical trial, investigating the bioavailability of RBT in combination with LPV/r.

## 1. Selection of patients:

1.1 Pre-screening

The recommended standard of care at Pham Ngoc Thach Hospital is for all newly diagnosed TB patients to be offered an HIV test, and CD4 lymphocyte count analysis if found to be HIV. Therefore, male or female individuals, aged 18 to 65 years (inclusive), with newly diagnosed pulmonary tuberculosis, who are HIV positive and have a CD4 less than or equal to $250/mm^3$ identified in Pham Ngoc Thach Hospital, will be eligible for the study.

1.2 Screening

Subjects attending the screening appointment who have volunteered to participate in the study will be requested to sign an informed consent form in accordance with international GCP standards. They will then undergo a full clinical evaluation and the following screening tests to determine eligibility for enrolment (blood samples taken will be approximately 25 ml):

- Full clinical examination, including ophthalmologic examination
- Full Blood Count (FBC) - Haemoglobin, platelet and differential white cell counts.
- Urea and electrolytes (U&E) - Sodium, potassium, urea, creatinine, bicarbonate
- Liver Function Tests (LFT) - Total bilirubin, aspartate and alanine aminotransferase, albumin, alkaline phosphatase
- Amylase
- HIV test
- CD4 count
- Urinary pregnancy test
- Urinalysis
- Chest X-ray (CXR)

1.3 Inclusion:

1.3.1. Inclusion Criteria:

Patients will be eligible for inclusion in the trial if they fulfil the following criteria:

i. Having a diagnosis of pulmonary tuberculosis defined as:

a) at least 2 sputum smears positive (equal to or exceeding grade 1) for AFB; or

b) 1 sputum smear positive for AFB and a chest radiograph (CXR) compatible with active tuberculosis; or

c) 1 sputum culture positive for *M. tuberculosis* and a CXR compatible with active tuberculosis

d) A CXR compatible with active tuberculosis and a clinical history suggestive of tuberculosis and 2 sputums smear negative for AFB

ii. Positive HIV antibody test and a CD4 lymphocyte count less than or equal to 250/$mm^3$. Weight>40 Kg.

iii. Having received no ART at all

iv. Having no grade 3 or 4 clinical or laboratory findings according to DMID tables.

v. If a female of childbearing age, having a negative pregnancy test on day of enrolment and willing to take appropriate contraceptive measures during the duration of the trial.

vi. Having a firm home address that is readily accessible

vii. Have a Karnofsky score Q≥ 80%

1.3.2. Non inclusion Criteria:

Patients who meet <u>any</u> of the following criteria will not be eligible for the study:

i. History of TB (within last 12 months) or MDR TB treatment (at anytime)

ii. Concomitant opportunistic infection (OI) requiring additional anti-infectious treatment

iii. A formal contraindication to any drug used in the trial

iv. Diabetes mellitus requiring drug treatment

v. Recreational drug or alcohol abuse

vi. History of drug hypersensitivity to TB or related medications

vii. Mental illness that could impair ability to give informed consent or result in poor adherence to trial protocol and therapy.

viii. Neutropenia (total neutrophil count < 1200 cells/L)

ix. Anaemia (haemoglobin <6.8 g/dL)

x. Requiring concomitant medications that may potentially interact with study drugs

xi. Liver Function Test > grade 2

xii. Pregnant or lactating women

xiii. Karnofsky score Q<80%

xiv. Any condition rendering the patient unable to understand the nature, scope, and possible consequences of this study and to provide consent.

Grading for the purposes of evaluating inclusion criteria will be according to the Division of Aids table for grading the severity of adult and paediatric adverse events (published December, 2004).

1.4 <u>Randomisation</u>

Participants will be randomized to receive one of the individual treatment arms on day of enrolment.. Randomization lists will be produced prior to the start of the trial, indicating a randomization number and

which treatment arm to assign to a participant. The code for each individual will be provided in a separate sealed envelope and assigned to the individuals in the order in which they are enrolled in the study.

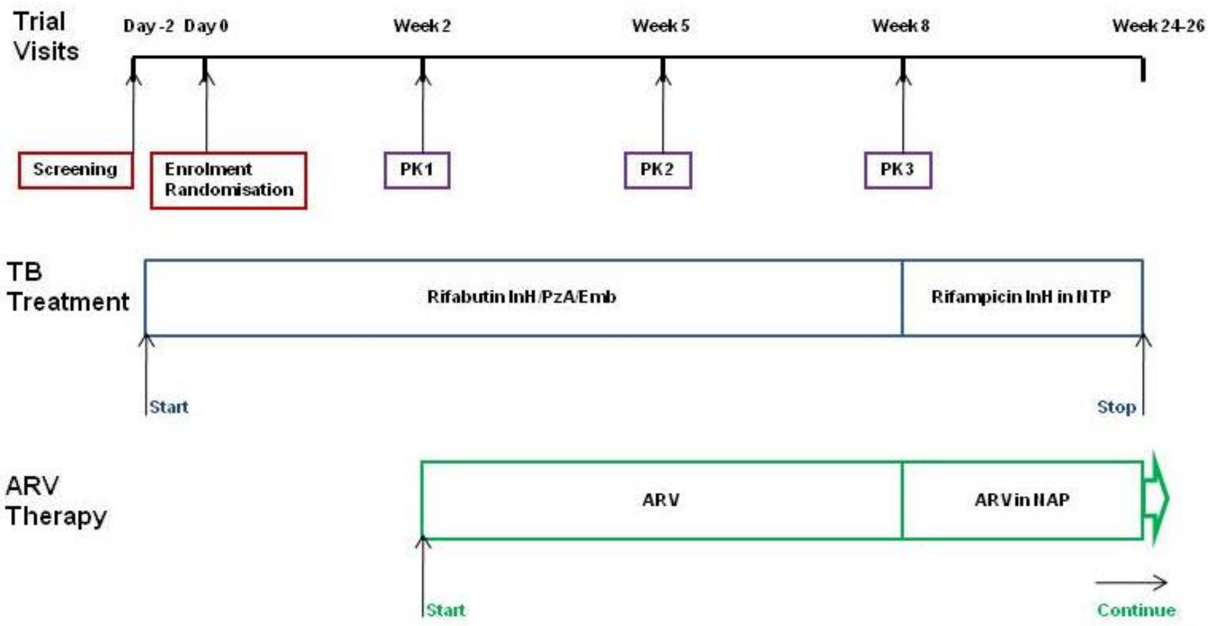


*Figure 1: Diagram showing the timing of the trial procedures relative to trial visits and treatments (Rmp= rifampin, Inh= isoniazid, PzA= pyrazinamide, Emb=ethambutol).*

## 2. Treatments under study

### 2.1 Treatment arms

All patients will be enrolled into the trial and started on RBT 300mg once a day (OD), in combination with standard doses of INH, PZA and EMB. After two weeks (representing the first 2 weeks of intensive phase of SCC), the first PK study (PK1) will be completed. Patients will then be switched to receive one of 2 combinations of RBT and ARV, as shown below, which they will take with other TB drugs, and the two subsequent PK studies (PK2 & 3) will be completed.

***Table 1**: The two arms of the trial and doses of drugs to be used initially. Arm A will switch dose to arm B dose after 5 weeks of therapy and arm B will switch to arm A dose after 5 weeks of therapy.*

| *Arm* | *ART* | *Rifabutin Dose* | | *Participants* |
|---|---|---|---|---|
| A | 2 NRTI + LPV/r (2 Tabs BD) | RBT | 150mg TPW | 16 |
| B | 2 NRTI + LPV/r (2 Tabs BD) | RBT | 150mg OD | 16 |

LPV/r = boosted lopinavir;

OD = once daily; BD = twice daily; TPW = three times per week

*Figure 2: Detailed trial timeline describing the allocation of treatments in the 2 arms of the trial, in relation with the intensive and continuation phases of TB therapy and randomization (times correspond to the number of weeks on treatment for the various combinations of drugs for tuberculosis). D4T = stavudine, 3TC = lamivudine, LPV/r = boosted lopinavir.*

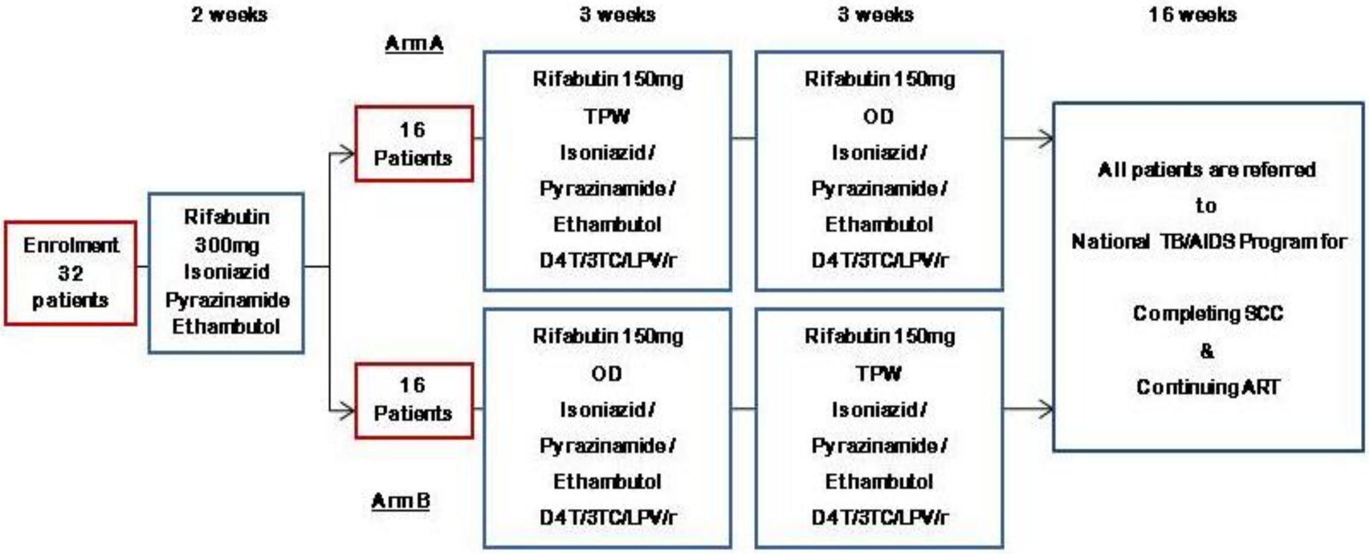


In each arm of the study, RBT is being taken at *two different doses* in combination with ARVs, using a *cross-over* design: Up to 32 patients will be randomized, of which half will start on the “A” dose and half on the “B” dose; after 3 weeks of therapy on SCC/ART, and following PK2 analysis, participants on the “A” dose will be switched to the “B” dose and vice-versa, in order to carry out PK3 analysis three weeks after the switch.

After the PK3, during the continuous phase of tuberculosis treatment, participants will be referred to National Tuberculosis Program and to National AIDS Program to be treated according to standard care. Rifabutin will be switched to rifampicin and lopinavir/rtv to efavirenz. Thus patients will be treated and followed according to recommendations from and by the National TB Program and by the National AIDS program An end study visit will be done at the end of the TB treatment between W24 and W26.

2.2. ARV selection:

LPV/r will be at a dose of 400 mg/100mg twice daily (film coated tablets). The NRTI backbone will be lamivudine (3TC) 150mg twice daily in combination with stavudine (d4T) 30mg twice daily. LPV/r has been selected as the protease inhibitor drug for evaluation in this project because it is the most widely available second-line drug, and has an excellent long-term efficacy and safety profile. The availability of the new film-coated tablets that do not require refrigeration is particularly attractive for eventual use in warm climate countries. Participants intolerant to d4T, and requiring a change of therapy, will be allowed to switch to zidovudine 300mg BD without withdrawal from the trial. If AZT or Tenofovir becomes the standard of care for first line treatment of HIV in Vietnam either of these drugs can be substituted for d4T. Intolerance to any of the other antiretroviral drugs necessitating their substitution will require that the participant is withdrawn from the trial.

2.3 RBT Dosing

The international guidelines for use of RBT in combination with ARVs is 150mg TPW or on alternate days with LPV/r (*17*, *18*). These guidelines are based on limited existing data, and there is still uncertainty if these doses are optimal. Co-administration of LPV/r with RBT is reported to result in large increases in RBT $AUC_{0-24}$(*22*) which supports a dose reduction of RBT to 150mg TPW. However these results are from a limited number of healthy volunteers, and rifamycins concentrations maybe lower in immunocompromised HIV positive individuals (*16*). Furthermore there are some recent anecdotal reports suggesting the 150mg intermittent dose of RBT is inadequate (*23*, *24*). Therefore 150mg RBT daily will also be evaluated in this proposal.

The development of acquired rifamycin resistance (ARR) in HIV positive tuberculosis patients treated with intermittent chemotherapy regimens has been reported with rifabutin-based regimens (*25*), and was associated with low CD4 lymphocyte counts (<100) and highly intermittent regimens (once or twice weekly). It is unknown if thrice weekly dosing with these agents would also favour the emergence of resistance. A recent retrospective study from USA found that ARR was also associated with intermittent rifampin regimens, suggesting that the emergence of resistance is a rifamycin class effect, rather than one restricted to the longer

acting agents rifabutin and rifapentine (*26*). Given these uncertainties this protocol has adopted daily rifabutin dosing (except for the low dose rifabutin in combination with LPV/r which will be thrice weekly) which is compatible with the Vietnamese TB control programme schedule of daily dosing.

**3. Outcome measures:**

3.1 Primary outcomes:

The primary study objective is to compare the pharmacokinetics of two different doses of RBT in combination with LPV/r, with the pharmacokinetics of RBT taken without ART in HIV infected patients with pulmonary TB and advanced immunodeficiency. The pharmacokinetics of RBT without ARV will be measured by the area under the curve ($AUC_{0-24}$), and compared to the $AUC_{0-24}$ for each of the RBT doses in combination with the ARV (and $AUC_{0-48}$ in case of the three times weekly LPV/r arm).

3.2 Secondary outcomes:

1. The pharmacokinetics of LPV/r in combination with two doses of RBT determined by the $AUC_{0-12.}$

2. The tuberculosis cure determined by sputum smear examination in line with the National TB Programme Guidelines

3. Proportion of participants with any grade 3 or 4 AE or SAE.

**4. Sample size**:

Based on the $AUC_{0-24}$ for RBT determined in previous studies (*19*) it was estimated that a sample size of 12 participants has a power of 80% to detect a 20% difference between the mean $AUC_{0-24}$ for the participants taking RBT without ARV and the $AUC_{0-24}$ for RBT combined with the various ARV. To provide a target of 12 evaluable patients in each arm (24 patients in total), because patients with low CD4 counts liable to be recruited into this study may have high mortality and morbidity, resulting in a high attrition rate, we propose a maximum of 32 patients may be enrolled (16 per arm). These additional subjects will allow a comparison of pharmacokinetic parameters between patients initiating high dose RBT with those initiating low dose RBT, and sufficient power to detect any sequence effect.

**5. Investigational plan:**

5.1 Trial preparation:

An initial preparative period of 4 months is planned for submission of the amended protocol to the ad-hoc institutions in Vietnam for approval (i.e. the Institutional Review Board and Ethical Review Committee at

PNTH, the Health Department of HCMC and the Ministry of Health of Vietnam). In parallel, additional staff will be recruited as appropriate, and all staff participating in the study will receive appropriate training (/re-training). This period will be used to prepare and print the Case Report Forms (CRF), draft the Standard Operating Procedures (SOP), and order and deliver drugs to the qualified pharmacist at Pham Ngoc Thach Hospital.

5.2 Trial Schedule

After this initial set-up, recruitment will be started and completed in 6 months. It is envisaged that an average of 5 patients per week will be a feasible recruitment rate. Follow up of patients will be until 2 weeks after tuberculosis treatment. The full PK analyses will be carried out after the PK3 day has been done for the last patient recruited, which will be approximately month 12 of the study, leaving four months for completion, writing up and publication (see table 2).

***Table 2:** Trial Schedule*

| | Screening<br>Day -2 | Visit 1<br>Day 0 | Visit 2<br>Week 2 | Visit 3<br>Week 5 | Visit 4<br>Week 8 | Visit 5<br>Week 24[6] |
|---|---|---|---|---|---|---|
| **Patient Information** | X | | | | | |
| **Informed Consent** | X | | | | | |
| **Inclusion/Exclusion** | X | X | | | | |
| **Randomisation** | | X | | | | |
| **Clinical History** | X | | | | | |
| **Physical examination** | X | X | X | X | X | X |
| **Adverse Event Screening** | | X | X | X | X | X |
| **Karnofsky score** | X | | | | | |
| **CD4 Count[3]** | X | | | | | X |
| **HIV antibody test** | X | | | | | |
| **HIV viral load[4]** | | | X | | | X |
| **FBC[1]** | X | | X | X | | X |
| **U and E[2]** | X | | | | X | |
| **LFT[2]** | X | | X | X | X | X |
| **Amylase[2]** | X | | | | X | |
| **Pregnancy Test** | X | | | | X | |
| **Urinalysis** | X | | | | X | |
| **Chest X-ray** | X | | | | X | X |
| **Sputum Smear and Culture** | X | | | | X* | X |
| **Susceptibility Testing** | X | | | | | X |
| **Pharmacokinetic analysis** | | | PK1 | PK2 | PK3 | |
| **Optional Pharmacogenomics sampling[5]** | | | X[5] | | | |
| **Start Rifabutin/INH/Ethamb/PZA** | | X | | | | |
| **Stop Rifabutin/INH/Ethamb/PZA & start Rifamp/INH** | | | | | X | |
| **Start D4T/3TC-LPV/rtv** | | | X | | | |
| **Stop LPV/rtv & start Efavirenz** | | | | | X | |
| **Stop Rifamp/INH** | | | | | | X |

[1, 2, 3.] The tests included are listed in section 1.2. The volumes of blood required for each set of tests are FBC: 4ml[1], Biochemical tests: 4ml[2], CD4 count: 4ml[3], Viral load: 8ml[4];

[5]Pharmacogenomics (optional and for patients who give specific consent): 8ml[5]

**[6]** last visit can be done between week 24 and week 26.

* Sputum smears in smear-positive PTB patients

5.3 Recruitment procedures:

Patients who are eligible for the trial will be recruited at the Outpatient unit in Pham Ngoc Thach (PNT) Hospital, where new cases of tuberculosis are diagnosed and receive their full treatment under direct observation (see Appendix 1). The appointed clinical trial nurse and counsellor will liaise with the doctors responsible for managing tuberculosis to identify patients who have been diagnosed with pulmonary tuberculosis and HIV, who have had a CD4 count analysis. Patients will be given full information on the objectives and methods of the trial. Patients will be given a copy of the information sheet to read before the screening appointment. If, at time of screening, patients present with an intercurrent opportunistic infection requiring specific concomitant treatment, they will not be included in the study.

Patients meeting eligibility criteria after screening will be enrolled in the trial and started on 300mg RBT in combination with INH, PZA, and EMB, corresponding to day 0 of the trial (visit 1).

5.4 Follow-up procedures:

In the Vietnamese TB Control Programme, patients come to District TB Units (DTU) every day to receive anti-TB drugs under the supervision of health workers. All patients involved in the study will come to the Outpatient Clinic at PNT Hospital to take their drugs, to receive specific training on HIV and TB as well as scheduled appointments. PNT Hospital will hold this organization according to the requirements of the study. Those who miss their scheduled appointment will be contacted within 24 hours or a home visit made to obtain information on their condition. Participants will be asked to return after 14 days

After fourteen days of RBT therapy, patients will return and will undergo the **first pharmacokinetic analysis (PK1)** as described below, as well as safety bloods and clinical evaluation. After completion of PK1 analysis, all participants will commence their randomized RBT-ART combination according to prior randomization.

1. After 3 weeks of ART, patients will complete a week 5 visit (visit 3) corresponding to **PK2** and then switch RBT doses. After a further 3 weeks of ART, patients will complete a week 8 visit corresponding to **PK3**. After completion of PK3, all participants will be referred to national program to complete SCC and continue ART according to standard program care. At the end of tuberculosis treatment, patients will have a blood taken for haematological and biochemical tests, HIV viral load and CD4 count - as well as a sputum for smear and microbiology. The results of these tests will be made available for the clinician in charge of the patient in the National Program.

2. Participants who have failed to achieve viral suppression (<250 copies/ml) will be offered to have an HIV genotypic resistance test performed in order to help clinician to propose an adapted ART second line.
3. All participants who give consent will participate in the pharmacogenomics sub-study.

5.5 Adverse Events (AEs) and Severe Adverse Events (SAEs):

Patients will be assessed for the development of AEs and SAEs at each scheduled clinic and PK visit, and at any unscheduled visits.

An SAE is defined as any untoward medical occurrence that:

- results in death
- is life threatening
- requires inpatient hospitalization or prolongation of existing hospitalization
- results in persistent or significant disability/incapacity
- Pregnancy
- is a congenital anomaly/birth defect

SAEs will be reported within 24 hrs of the investigator becoming aware of the event. The report will be made by fax or e-mail to Dr Dominique Lagarde, or designee, at The Union Head Office in Paris, and a full report will be subsequently completed for each SAE. Dr Dominique Lagarde will be responsible for reporting all SAEs to ANRS within 7 days. Full procedures for reporting SAE's are outlined in Appendix 2.

At enrolment (day 0), a full eye examination will be carried out, including assessment of visual acuity, pupillary reflexes, eye movements and fundoscopy. Focused questions will be asked at screening and each visit to determine if eye pain, eye redness, sensitivity to light or blurred vision is present and an external eye examination will be carried out to facilitate the early detection of uveitis (a recognized AE associated with RBT). Patients will be warned to report any ocular symptoms. Patients reporting eye symptoms will be referred for full ophthalmologic examination.

Each participant will also be asked about arthralgia at each visit. Regular laboratory test monitoring will allow early detection of neutropenia, renal insufficiency, hepatitis. Patients will be encouraged to report to the clinic at any time during the follow-up period if they feel unwell, in order to maximise the capture of AEs. Any death will be registered and all efforts will be made to identify the likely cause(s) of death. AEs occurring during the study will be evaluated by the investigator and graded according to the Division of Aids table for grading the severity of adult and paediatric adverse events. The relationship between the trial medications and each AE/SAE will be investigated and graded with regard to likelihood of causality.

In the event of hospitalization the trial physician will liaise directly with the appropriate medical staff. The designated trial physician assesses all participants who are inpatients on a regular basis. Particular attention will be given to the detection and management of IRIS. Other possibilities (drug fever, treatment failure, TB recurrence, drug resistance, opportunistic infection) need to be ruled out with all appropriate evaluations including multiple blood cultures, urine, chest x-ray, sputa testing and other procedures. If IRIS is suspected, symptomatic therapy (anti-inflammatory agents) will be initiated at the discretion of the study clinician. A separate IRIS reporting form will be used to ensure adherence to a standardized diagnosis and treatment protocol.

### 5.6 Microbiology procedures:

At screening and at the end of the trial sputum will be tested by smear examination and culture, using Lowenstein-Jensen solid medium**.** Baseline isolates will be preserved in glycerol enriched media and their drug susceptibility compared with any isolate obtained at the end of therapy. Patients diagnosed with multidrug resistant tuberculosis (MDR-TB) will be withdrawn immediately from the trial and referred for initiation of MDR-TB treatment to the appropriate structure as soon as the second line treatment is available in Viet Nam.

### 5.7 Immuno-virological procedures:

Blood samples will be collected at time of screening for a confirmatory HIV test and CD4 count. These tests will be performed at Pasteur Institute HCMC. In addition, samples for HIV viral load quantification will be tested using a commercial assay. Aliquots of all samples will be preserved for subsequent evaluation of genotypic resistance to ART.

### 5.8 Pharmacological procedures and assays:

Participants will be admitted to the clinical pharmacokinetic facility at Pham Ngoc Thach Hospital for the evening prior and after PK sampling on 3 separate occasions and meals and snacks will be standardized between the three PK studies. **PK1** will be conducted once steady state dosing has been established. This will be achieved by keeping patients on RBT during the first 2 weeks of the SCC intensive phase and carrying out PK analysis after two weeks of the intensive phase therapy have been completed. **PK2** and **PK3** will be completed 3 and 6 weeks after PK1, at which time steady state dosing of the ARV regimen has been established. Blood samples will be drawn into lithium-heparin coated tubes at each of the times after drug administration illustrated in Table 3. Five millilitres of blood will be collected for RBT assays alone and eight millilitres for RBT and ARV assays.

***Table 3:*** *Table showing the sampling times for each arm at each pharmacokinetic visit*

| | PK 1 | PK2 | | PK3 | |
|---|---|---|---|---|---|
| **Visit number** | **2** | **3** | | **4** | |
| **Arms** | **A, B** | **A** | **B** | **A** | **B** |
| **Sampling relative to morning dose** | **Assays** | | | | |
| **-0.5 to 0 hrs** | RBT | RBT, LPV | RBT, LPV | RBT, LPV | RBT, LPV |
| **2 hrs** | RBT | RBT, LPV | RBT, LPV | RBT, LPV | RBT, LPV |
| **3 hrs** | RBT | RBT, LPV | RBT, LPV | RBT, LPV | RBT, LPV |
| **4 hrs** | RBT | RBT, LPV | RBT, LPV | RBT, LPV | RBT, LPV |
| **5 hrs** | RBT | RBT, LPV | RBT, LPV | RBT, LPV | RBT, LPV |
| **6 hrs** | RBT | RBT, LPV | RBT, LPV | RBT, LPV | RBT, LPV |
| **8 hrs** | RBT | RBT, LPV | RBT, LPV | RBT, LPV | RBT, LPV |
| **12 hrs** | RBT | RBT, LPV | RBT, LPV | RBT, LPV | RBT, LPV |
| **24 hrs** | RBT | RBT | RBT | RBT | RBT |
| **48 hrs** | | RBT | | | RBT |
| **Total Blood Sampled** | 45 ml | 50 ml | 45 ml | 45 ml | 50 ml |

NB: For the drug assays RBT = rifabutin. LPV= Lopinavir.

Intensive sampling will be used over the dosing interval in order to adequately characterize the pharmacokinetic measures of importance (AUC, Cmax). The samples will be immediately stored in crushed ice for a maximum of 1 hour, until centrifugation and separation of plasma on site. Separated plasma will be transferred into 2 separate polypropylene (microcentrifuge) tubes, each containing approximately 1 ml of plasma before immediate storage in the Pham Ngoc Thach laboratories at -80C. The exact times of specimen collection will be recorded in the case record forms. Aliquots of the plasma samples will be transferred on dry ice to the Department of Biological Analysis at the Pasteur Institute HCMC by door-to-door transport, for PK analyses. The concentrations of rifabutin, its major metabolite 25-deacetylrifabutin and LPV/r will be determined by High Pressure Liquid Chromatography (HPLC). Validated methods will be developed according to GLP in conjunction with Kremlin-Bicêtre Hospital Pharmacology laboratory in Paris, and a QA procedure will be set between Pasteur Institute HCMC and the University of Cape Town Clinical Pharmacology Division.

5.9 <u>Pharmacokinetic analysis:</u>

On each series of drug plasma concentrations, the following pharmacokinetic parameters will be determined, using the software package WinNonLin (Pharsight Corporation, California).

- Area under the curve for plasma concentration vs. time, until the last sampling time after dosing (AUCt)

- Peak plasma concentration ($C_{max}$)
- The trough plasma concentration at the last time of sampling, immediately before the next dose.
- Time to peak plasma concentration ($T_{max}$).
- Half-life ($T_{½}$).

The PK measures will be summarized using parametric or nonparametric methods (as appropriate). For RBT, the results from PK1 will be compared to PK2 and PK3, respectively. PK2 and 3 will be compared for all the drugs. The significance of differences between the pharmacokinetic measures will be determined using the Student t-test (parametric data) and the Wilcoxon signed rank test (nonparametric data). Bioequivalence analysis will be conducted using ANOVA for the AUC and Cmax values. The geometric mean ratios and their 90% confidence intervals will be reported. In addition the inter-individual and intra-individual variability will be evaluated for the PK measures. No interim PK analyses are planned. In addition, potential polymorphisms in specific enzymes involved in drug metabolism will be investigated.

5. 10 Pharmacogenomics samples

DNA analysis will be performed on whole blood in order to determine the polymorphisms of the cytochrome P450 and other genetic loci implicated in drug metabolism. These analyses will be carried out by the Pharmacology Unit of Hospital Kremlin-Bicêtre in Paris in collaboration with other laboratories where necessary. Techniques for agnostic screening of genome wide loci using microarrays, automated DNA sequencing or other technologies may also be deployed. Shipment of samples will be the responsibility of Pasteur Institute, Ho Chi Minh City and the Pharmacology Unit of Hospital Kremlin-Bicêtre in Paris.

5.11 Storage of samples

Aliquots of plasma will be stored at -80 degrees in the Department of Biological Analysis at Pasteur Institute, HCMC. Microbiological samples will be stored in the Department of Microbiology at Pham Ngoc Thach Hospital for 2 years.

All efforts will be made in order to upgrade the Pasteur Institute laboratory to perform the pharmacokinetics analysis of lopinavir/ritonavir, and rifabutin/metabolite. This will be done through appropriate training and transfer of technology. Human and non human samples will be exchanged between the Pharmacology Unit at University Hospital Kremlin-Bicêtre in Paris (France) and Pasteur Institute in Ho Chi Minh City, to perform the necessary quality controls. If, for exceptional and unpredictable reasons, Pasteur Institute is unable to carry out the PK analyses by the end of follow-up

of the last patient included in the study, plasma samples will be transferred to the Pharmacology Unit of Hospital Kremlin-Bicêtre in Paris in order to perform the pharmacokinetics analyses.

5.12 Provisional dates:

| | |
|---|---|
| Submission to authorities: | 15 April 2008 |
| Full approval received: | 23 March 2009 |
| Approval Vietnamese Ministry of Health: | 13 October 2008 |
| Re-approval Vietnamese Ministry of Health: | 21 April 2010 |
| Start recruitment: | 25 June 2010 |
| Enrolment interruption: | 19 October 2010 |
| Submission protocol version 4. 2: | 20 April 2011 |
| Expected approval by Vietnamese Ministry of Health: | 15 June 2011 |
| Start enrolment: | 15 August 2011 |
| Finish enrolment: | 15 February 2012 |
| Complete follow-up: | 15 August 2012 |
| Complete analysis and reporting: | 15 November 2012 |

**6. Trial management:**

A *Trial management group* (TMG) will comprise the investigators of the four institutions involved in the study. The TMG will be responsible for supervising all activities related to the preparation and the conduct of the trial, and take decisions to solve emerging problems. The TMG will meet regularly and ad-hoc teleconference meetings will take place as appropriate.

A fully independent *Data and Safety Monitoring Committee (DSMC)* will be set-up, composed of at least a chairman, a clinician and a statistician, all external to the trial team. Its role is to provide advice on all aspects of the trial and make specific recommendations, particularly on ethics and safety issues.

- *Study monitoring:* The investigator in Vietnam will maintain all study documentation, including the Investigator's Study File and the subjects' clinical source documents, as requested under GCP requirements. In accordance with GCP procedures, a trial monitor will be appointed to review protocol compliance, data collection and conduct site visits to check the progress of the study, review data collected and conduct source document verification. This will also ensure that data are authentic, accurate and complete, that safety rights of subjects are being protected, and that the study is being conducted in accordance with the currently approved protocol (and any amendments), along with GCP

and all applicable regulatory requirements. This study monitor will be regularly supervised by the Clinical Coordinator of the study.

- *Data management:* Case Report Forms will be prepared prior to the trial and piloted. A Master database will be set up and a unified data entry system will be used, with a unique identifying number for each patient recruited in the trial. Pham Ngoc Thach Hospital will be responsible for regular data entry, using double entry with built-in checks.

- *Confidentiality:* All study procedures will be conducted in private, and every effort will be made to protect participant privacy and confidentiality. All participant information, including forms, lists, logbooks, appointment books and any other listings that link participant ID numbers to other identifying information will be stored in locked filing cabinets in areas with limited access. Administrative forms, laboratory and other reports, will be identified only by the randomisation number. All databases will be secured with password-protected access systems. Participants' study information will not be released without their written permission, except as necessary for monitoring of the study conduct and data collection.

**7. Ethical aspects:**

This study will be conducted in accordance with "Good Clinical Practice" (GCP) and all applicable regulatory requirements, including the Declaration of Helsinki (Edinburgh 2000 revision). Particular attention is given to the fact that the project passes ethical approval. The protocol will be submitted to the ad-hoc Ethics Committee in Vietnam (Ethical Review Board of Pham Ngoc Thach and Ethical Review Board of Ministry of Health) and in France (The Union Ethics Advisory Group). An informed consent form will be required to be signed by all potential subjects. These forms will describe clearly the potential benefits and risks of participating in the study, and be prepared in non-technical English. They will be translated into Vietnamese. If the patient is unable to read, an independent witness will be present to ensure that the patient receives complete information. It will be made clear that the person may freely withdraw consent at any time. The informed consent process will cover all elements of informed consent required by research regulations.

**8. Procurement of drugs and study material:**

The drugs will be ordered as much as possible using generic products and will be delivered to the patients recruited in the study free of charge. Purchase of drugs at commercial value will have a high cost, so various companies and institutions are currently being approached in order to negotiate either donations of drugs or low-cost supply of investigational products. The Union will procure the antiretroviral drugs, except Lopinavir/r, that will be provided by its manufacturer, Abbott

Pharmaceuticals. Rifabutin will be obtained from SERB pharmaceutical company. Quality control analyses will be provided for all these drugs. Other antituberculous drugs will be provided by the National Tuberculosis Program. Reagents and other material for analysis of PK samples will be imported as appropriate.

## 9. Trial Site and collaborating institutions:

*9.1 Outpatient Department, Pham Ngoc Thach Hospital.*

Pham Ngoc Thach Hospital (PNTH) is the South Coordinator and Principal Investigator of the study in Vietnam. PNTH will be responsible for the general performance of the study in Vietnam and will contribute to all activities pertaining to the setting-up of the study in Vietnam. Patients will be recruited at the Outpatient Department of PNTH, where all due procedures for recruitment will be performed. PNTH is the highest class referral hospital specialized in TB and Lung diseases and technically responsible for TB and Lung Disease in the South of Vietnam and it has strong reputation in communities regarding TB and TB/HIV control. The team at the PNTH will be responsible for selecting, informing and recruiting patients for the PK study. Selected patients will be given full treatment as appropriate and will be closely monitored for the whole duration of the study. PNTH will be responsible of general patients' care as well as all due microbiology and biological analyses. PNTH will also be responsible for collection of PK samples on PK days, and for their processing and transfer to Pasteur Institute, HCM City. The team at PNTH will also be responsible for keeping all due source documents (informed consent forms, case registration forms, etc… according to GCP requirements) as well as for data entry and data transfer for analysis.

*9.2 Department of Biological Analysis, Pasteur Institute, Ho Chi Minh City:*

Pasteur Institute is co-investigator of the study in Vietnam, and the local ANRS Representative. Pasteur Institute will be responsible for laboratory support for HIV related analysis, as indicated in the protocol: CD4 count, HIV RNA, HIV genotyping, HCV serology, HBV serology. Pasteur Institute will be responsible for the Pharmacokinetic analyses, using an HPLC machine, with assistance from the Department of Pharmacology, Hôpital Kremlin Bicêtre, in Paris (France) for technology transfer and External quality assessment. Cross-validation will be with the Pharmacology Laboratory at University of Cape Town (South Africa).

*9.3 Department of Pharmacology, Hospital Kremlin Bicêtre, Paris:*

This is the Laboratory of Therapeutic Drug Monitoring and Pharmacokinetics of Department of Clinical Pharmacy, at the University Teaching Hospital of Kremlin Bicêtre, Paris (France). ARV drugs are measured in plasma by HPLC assays according to good laboratory practice. This wrote re would be responsible for capacity building and technology transfer to the Pasteur Institute in Ho Chi Minh City, and will ensure also external quality assessment.

*9.4 International Union against Tuberculosis and Lung Disease, Paris, France*. The Union is the North Coordinator and Principal Investigator of the Rifabutin PK Study in France. The Union will be responsible for the general coordination and supervision of the study, review of data, data analysis and collaboration with South Africa PK teams. The Union will contribute to the setting up of the study in Vietnam in collaboration with Vietnamese collaborators, including protocol amendments, design of CRFs and SOPs, trial supervision and monitoring and data analysis.

**10. Publication policy**

The principal investigators will prepare in a timely fashion a manuscript for publication of the principal results of this study in collaboration with the co-investigators. Authorship of this article will acknowledge the contributions of the Pham Ngoc Thach Hospital, Pasteur Institute HCMC, Hopital Kremlin Bicêtre and IUATLD. Other publications or public presentations of data derived from this study will be managed by the principal investigators in full consultation with the co-investigators. These publications and presentations will acknowledge the contributions of Pham Ngoc Thach Hospital, Pasteur Institute HCMC, Hopital Kremlin Bicêtre and IUATLD. The ANRS will be cited as sponsor of this trial in all publications or presentations of results derived from this study.

## References


1. Vermund SH, Yamamoto N. Tuberculosis (Edinb). 2007;87 Suppl 1:S18-25.
2. Laserson KF, Wells CD. Bull World Health Organ. 2007;85:377-81; discussion 382-6.
3. World Health Organization (2006) Global tuberculosis control: surveillance, planning, financing. WHO report 2006. Geneva, Switzerland: World Health Organization.
4. Louie JK,et al., Int J STD AIDS. 2004;15:758-61
5. HIV/AIDS epidemiological fact sheet in Viet Nam, WHO, available at http://www.who.int/globalatlas/predefinedReports/EFS2006/EFS_PDFs/EFS2006_VN.pdf (last accessed on January 2008).
6. Quy HT, et al., AIDS. 2002;16:931-2.
7. L. Aaron *et al.*, *Clin Microbiol Infect* **10**, 388-98 (May, 2004).
8. A. Kwara, T. P. Flanigan, E. J. Carter, *Int J Tuberc Lung Dis* **9**, 248-57 (Mar, 2005).
9. M. Niemi, J. T. Backman, M. F. Fromm, P. J. Neuvonen, K. T. Kivisto, *Clin Pharmacokinet* **42**, 819-50 (2003).
10. W. Manosuthi *et al.*, *Aids* **20**, 131-2 (Jan 2, 2006).
11. J. Oliva *et al.*, *Aids* **17**, 637-8 (Mar 7, 2003).
12. C. J. la Porte *et al.*, *Antimicrob Agents Chemother* **48**, 1553-60 (May, 2004).
13. G. Jourdain *et al.*, *N Engl J Med* **351**, 229-40 (Jul 15, 2004).
14. C. Laurent *et al.*, *Emerg Infect Dis* **12**, 1001-4 (Jun, 2006).
15. S. Schwander *et al.*, *Tuber Lung Dis* **76**, 210-8 (Jun, 1995).
16. H. McIlleron *et al.*, *Antimicrob Agents Chemother* **50**, 1170-7 (Apr, 2006).
17. J. F. Delfraissy, *Recommandations du groupe d'experts* http://www.sante.gouv.fr/htm/actu/delfraissy/index.htm (2002).
18. DHHS Panel, *http://aidsinfo.nih.gov/Guidelines/GuidelineDetail.aspx?MenuItem=Guidelines&Search=Off&GuidelineID=7&ClassID=1* (2006).
19. M. Weiner *et al.*, *Clin Infect Dis* **41**, 1343-9 (Nov 1, 2005).
20. Boehringer Ingelheim, *http://us.viramune.com/hcp/product_desc.jsp* (2006).
21. M. K. Jordan *et al.*, *Antimicrob Agents Chemother* **44**, 2170-2 (Aug, 2000).
22. Abbott, *www.kaletra.com/* (2006).
23. H. Khachi, D. Ladenheim, C. Orkin, N. Abdelmagid, K. Scott, *HIV Med* **7(Suppl. 1)**, (abstract no. O12) (2006).
24. S. Bonora *et al.*, *Antiviral Therapy* **8**, S427-S428 (2003).
25. W. Burman *et al.*, *Am J Respir Crit Care Med* **173**, 350-6 (Feb 1, 2006).
26. J. Li, S. S. Munsiff, C. R. Driver, J. Sackoff, *Clin Infect Dis* **41**, 83-91 (Jul 1, 2005).

27. *Estrela RC, Ribeiro FS, Barroso PF, et al. ABCB1 polymorphisms and the concentrations of* lopinavir and ritonavir in blood, semen and saliva of HIV-infected men under antiretroviral therapy. Pharmacogenomics 2009; 10 (2) : 311-8
28. Haas DW. Will pharmacogenomic discoveries improve HIV therapeutics? Topics in HIV Medicine 2005; 13 (3): 90-95

29. Haas, D. W., H. J. Ribaudo, R. B. Kim, C. Tierney, G. R. Wilkinson, R. M.Gulick, D. B. Clifford, T. Hulgan, C. Marzolini, and E. P. Acosta..Pharmacogenetics of efavirenz and central nervous system side effects: an Adult AIDS Clinical Trials Group study. AIDS 2004;18:2391-2400.

30. Hiratsuka, M., Y. Takekuma, N. Endo, K. Narahara, S. I. Hamdy, Y. Kishikawa, M. Matsuura, Y. Agatsuma, T. Inoue, and M. Mizugaki. Allele and genotype frequencies of CYP2B6 and CYP3A5 in the Japanese population. Eur J Clin Pharmacol. 2002;58:417-421.

31. Iatsimirskaia E, Tulebaev S, Storozhuk E et al., Metabolism of rifabutin in human enterocytes and liver microsomes. Clin Pharmacol Ther 1997;61:554-562

32. Loeuillet C, Weale M, Deutsch S et al., Promoter polymorphisms and allelic imbalance in ABCB1 expression. Pharmacogenet Genomics 2007; 17 (11): 951-9

33. Lubomirov R, di Iulio J, Fayet A, Colombo S, Martinez R, Marzolini C, Furrer H, Vernazza P, Calmy A, Cavassini M, Ledergerber B, Rentsch K, Descombes P, Buclin T, Decosterd LA, Csajka C, Telenti A; Swiss HIV Cohort Study. ADME pharmacogenetics: investigation of the pharmacokinetics of the antiretroviral agent lopinavir coformulated with ritonavir. Pharmacogenet Genomics. 2010;20(4):217-30.

34. Ma Q, Brazeau D, Zingman BS, et al. Multidrug resistance 1 polymorphisms and trough concentrations of atazanavir and lopinavir in patients with HIV. Pharmacogenomics 2007; 8 (3): 227-35

35. Saitoh A, Singh KK, Powell CA, et al. An MDR1-3435 variant is associated with higher plasma nelfinavir levels and more rapid virologic response in HIV-1 infected children. AIDS 2005; 19 (4) : 371-80

36. Telenti A, Zanger UM. Pharmacogenetics of anti-HIV drugs. Annu Rev Pharmacol Toxicol. 2008;48:227-56. Review

37. Tirona RG, Kim RB.. Nuclear receptors and drug disposition gene regulation. *J Pharm Sci* 2005;94:1169-86

38. Veiga, M. I., S. Asimus, P. E. Ferreira, J. P. Martins, I. Cavaco, V. Ribeiro, T. N. Hai, M. G. Petzold, A. Bjorkman, M. Ashton, and J. P. Gil. Pharmacogenomics of CYP2A6, CYP2B6, CYP2C19, CYP2D6, CYP3A4, CYP3A5 and MDR1 in Vietnam. Eur J Clin Pharmacol. 2009;**65:**355-363.

**Appendix 1**

## Description of sites

**1. Pham Ngoc Thach Hospital (PNTH)** is the highest class referral hospital, specialized in TB and Lung diseases. It is technically responsible for diagnostic and management of TB and Lung Diseases in the South of Vietnam, and it has strong reputation in communities regarding TB and TB/HIV control. The hospital has 700 beds for in-patients and 900 beds for out-patients. Nowadays, there are 7 functional offices, 22 wards for tuberculosis and lung disease, 2 wards for TB/HIV with more than 100 beds, and para-clinical services. The number of patients attending PNTH consultations for diagnosis and treatment is about 500 every day. The hospital has a good TB patient follow-up system. There are also VCT and OPC units for co-infected TB/HIV patients. These units are responsible for more than 500 HIV patients, 200 of them being on ARV treatment. PNTH staff is experienced in clinical practice as well as in research. Research team members have training in adhering to GCP and GLP guidelines and have large experience with clinical trials such as:

- Fixed dose combinations versus lose tables in TB (IUATLD Study C)
- Corticoids in TB meningitis (Oxford Clinical Unit)
- Immediate *vs.* deferred antiretroviral therapy in HIV+ TB meningitis, etc…

The immunology laboratory performs ELISA tests for HIV serology, tumour markers, hepatitis B and C serology, CD4/CD8 counts etc. It is capable of separating and freezing blood cells. The laboratory has 1 MD PhD level staff, 2 biologists and 20 ancillary staff.

The National Mycobacterial Reference Laboratory at PNTH performs 770 sputum smear examinations, 70 mycobacterial cultures and 15 tuberculosis drug susceptibility tests on average per day. Tests done include smear examination (direct and concentrated), culture drug susceptibility testing, RFLP typing and spoligotyping. The laboratory has 2 MD level staff, 1 BSc level staff, 1 MD PhD level staff, 3 biologists and 40 ancillary staff. Available equipment includes class 2 safety cabinets, centrifuges, incubators, BACTEC-MGIT and PCR equipment, a Biorad camera and -70°C freezers.

**2. Department of Biological Analysis, Pasteur Institute, Ho Chi Minh City:**

Pasteur Institute Ho Chi Minh City , 167 Pasteur Street, 8 Ward, 3 District, Ho Chi Minh City.

This is a summary of functions and activities of Pasteur Institute HCM, Vietnam in relation with the study theme. The detailed description is found in annex.

**Functions**

1) Research on medical biology, microbiology, immunology, epidemiology on infectious diseases.
2) Outbreak investigation and epidemic control (cholera, typhoid fever, dengue haemorrhagic fever, plague, Japanese encephalitis B…) and national health programs management:
3) Vaccine Production (BCG vaccine, rabies vaccine) and diagnostic bio-product.
4) Postgraduate and continued training.
5) Health services (Clinical Laboratory Analyses, Immunization and Dispensary services).
6) International collaboration for scientific research and training.

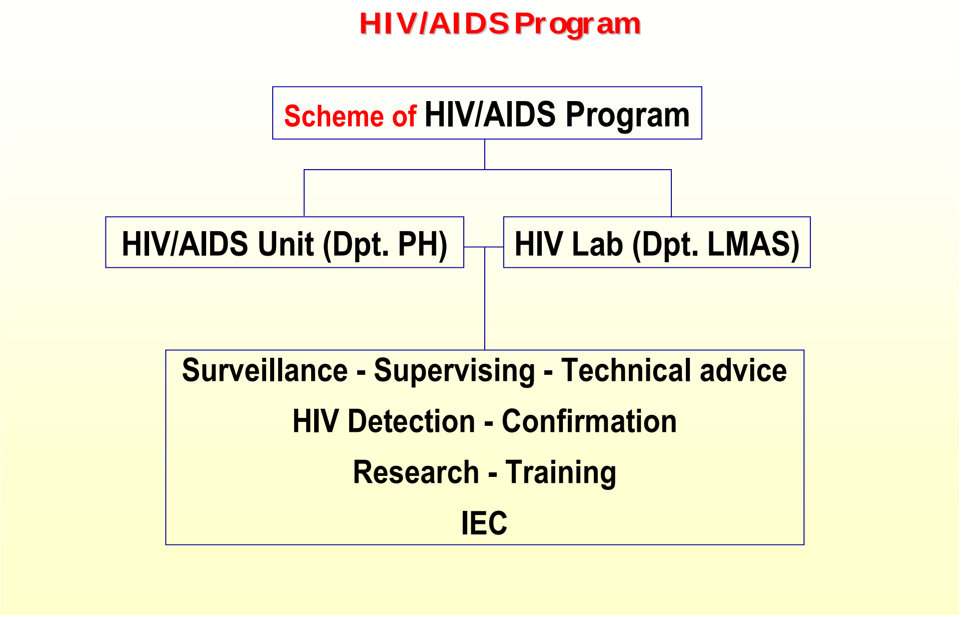


**Role of HIV/AIDS Program**

1. Surveillance
2. Information, education and communication
    - Preventive and supportive counselling on HIV/AIDS.
    - Publishing quarterly AIDS Bulletin
    - IEC materials
3. Lectures, training courses, workshops, seminars and meetings
4. HIV/AIDS related studies on epidemiology, biology and behaviour
5. Basic studies
6. Interventions

**3 Departments of Pharmacology, Hopital Kremlin Bicêtre, Paris***:*

This is the Laboratory of Therapeutic Drug Monitoring and Pharmacokinetics of Department of Clinical Pharmacy, Pharmacie CHU Bicêtre, Le Kremlin Bicêtre 94270, FRANCE. ARV drugs are measured in plasma by HPLC assays according to good laboratory practice.

*Main recent publications:*

1: Teicher E, Vincent I, Bonhomme-Faivre L, Abbara C, Barrail A, Boissonnas A, Duclos-Vallée JC, Taburet AM, Samuel D, Vittecoq D. Effect of Highly Active Antiretroviral Therapy on Tacrolimus Pharmacokinetics in Hepatitis C Virus and HIV Co-Infected Liver Transplant Recipients in the ANRS HC-08 Study. Clin Pharmacokinet. 2007;46(11):941-52.
2: Panhard X, Legrand M, Taburet AM, Diquet B, Goujard C, Mentré F; the Cophar 1 - ANRS 102 Study Group. Population pharmacokinetic analysis of lamivudine, stavudine and zidovudine in controlled HIV-infected patients on HAART. Eur J Clin Pharmacol. 2007 Nov; 63(11):1019-1029. Epub 2007 Aug 11.

3: Barrail A, Le Tiec C, Paci-Bonaventure S, Furlan V, Vincent I, Taburet AM. Determination of amprenavir total and unbound concentrations in plasma by high-performance liquid chromatography and ultrafiltration. Ther Drug Monit. 2006 Feb;28(1):89-94.
4: Goujard C, Legrand M, Panhard X, Diquet B, Duval X, Peytavin G, Vincent I, Katlama C, Leport C, Bonnet B, Salmon-Céron D, Mentré F, Taburet AM; COPHAR1-ANRS 102 Study Group. High variability of indinavir and nelfinavir pharmacokinetics in HIV-infected patients with a sustained virological response on highly active antiretroviral therapy. Clin Pharmacokinet. 2005;44(12):1267-78.

5: Panhard X, Goujard C, Legrand M, Taburet AM, Diquet B, Mentré F; COPHAR 1-ANRS study group. Population pharmacokinetic analysis for nelfinavir and its metabolite M8 in virologically controlled HIV-infected patients on HAART. Br J Clin Pharmacol. 2005 Oct;60(4):390 403.
6: Taburet AM, Piketty C, Chazallon C, Vincent I, Gérard L, Calvez V, Clavel F, Aboulker JP, Girard PM. Interactions between atazanavir-ritonavir and tenofovir in heavily pretreated human immunodeficiency virus-infected patients. Antimicrob Agents Chemother. 2004 Jun; 48(6):2091-6.
7: Taburet AM, Raguin G, Le Tiec C, Droz C, Barrail A, Vincent I, Morand-Joubert L, Chêne G, Clavel F, Girard PM. Interactions between amprenavir and the lopinavir-ritonavir combination in heavily pre-treated patients infected with human immunodeficiency virus. Clin Pharmacol Ther. 2004 Apr; 75(4):310-23.

**4. International Union against Tuberculosis and Lung Disease, Paris, France**. The International Union against Tuberculosis and Lung Disease (The Union) is a not-for-profit, non-governmental organization located in Paris (France), whose mission is “the prevention and control of tuberculosis and lung diseases on a worldwide basis, with a particular emphasis on low-income countries”. Over the last decade, The Union has expanded widely and, in addition to TB control and prevention, has taken a leading role in activities relating to lung health and TB/HIV care and prevention. The Clinical Trial Division (CTD) is coordinating trials of new drugs and/or combined regimens for the treatment of tuberculosis through an international network of clinical trial centres working up to GCP standards. The CTD works in collaboration with scientists and experts from reputed institutions worldwide (Medical Research Council, UK; Institute of Tropical Medicine, Antwerp, Belgium; London School of Hygiene and Tropical Medicine, University of New Mexico, USA; Institut de Recherche pour le Développement, France, etc.).

**Appendix II:**

**Serious Adverse Events reporting Procedure for ANRS Sponsored Research**

**in Developing countries**

This procedure defines the modes of Serious Adverse Events reporting for research in developing countries in order to comply with the directives of the International Conference for Harmonization (ICH4): "Clinical Safety Data Management definitions and standards for expedited reporting" (October 27, 1994).

**Serious Adverse Events definition:**

An **adverse event** refers to any harmful manifestation occurring in a patient or a clinical trial participant, whether this manifestation is related or not to the study drug(s).

A **serious Adverse Event** (SAE) refers to all adverse event that result in any of the following:

- death,
- immediate life-threatening,
- significant disability/incapacity (temporarily or permanently),
- inpatient hospitalization or prolongation of existing hospitalization,
- congenital anomaly/birth defect on descendant.

**An adverse event can be « *potentially serious* » if not included in the above definition when considered as serious according to the medical judgment of the investigator (ex: some biological anomalies) or if it demands preventive intervention to avoid that it becomes serious. This type of event should also be reported.**

SAE reporting to ANRS

Events to be reported must comply with the two following conditions:

- **Be Serious** (cf. above)
- **Be likely related to the research**: meaning that *"their onset cannot reasonably be attributed to an independent cause from the research condition"*. This notion includes the study drugs, diagnosis tests and all the constraints linked to the research.

Some SAEs not complying with this definition can be described in the research protocol: they should also be reported.

**Assessment of severity**

***For adults:*** The severity of Serious Adverse Events is assessed according to the "ANRS Toxicity Table For Grading Severity Of HIV+ Adult Adverse Events" (annex 1)

***For children:*** The severity is assessed according to the 2 NIH tables (before and after 3 months of age) « Division of AIDS Toxicity Table for Grading Severity of Paediatric Adverse Experiences – April 1994 » (annex 1).

Forms

The forms to be used are:

- The initial report form, for the first SAE

- The follow-up report form in two cases :
  - To complete the initial report if the investigator has new information on the SAE diagnosis, its evolution to recovery or aggravation (possibly to death), or the causal relationship to the study drug.
  - To answer to information asked by ANRS.

Forms, first validated by the ANRS Pharmacovigilance Department may be exceptionally used.

**Reporting schedule:**

A SAE should be reported to ANRS by the investigator **as soon as he is aware of it** and at least:

- Within 7 calendar days if death, life-threatening event or complementary information required by ANRS.
- Within 15 calendar days for the other cases.

**Period of reporting:**

During the duration of the trial and up to one month after the end of the trial: all SAE and pregnancies.

Anytime after the completion of the trial: all events suspected to be related to the study drugs (e.g.: congenital abnormalities).

**Reporting to National Authorities:**

Investigators should report SAE to their National Authorities according to the national regulations. The mode of reporting should be described in the study protocol.

**Reporting to Pharmaceutical companies:**

If a contract is signed between ANRS and a pharmaceutical company for the study drug supply, SAE reporting to the company should be done by the investigators according to the terms of the contract.

Particular case: Pregnancies

Pregnancies occurring after inclusion in the trial should be systematically reported to ANRS on the pregnancy form (annex 4) within 15 calendar days following the investigators awareness. The medical surveillance of the women and their children should be reinforced: a particular attention must be given on serious pathology occurring during pregnancy and congenital abnormalities. An SAE initial report form should be filled in if any anomaly is detected.

## ANRS SAE REPORTING - SUMMARY TABLE

| Case n° | What? | Which form? | When? |
|---|---|---|---|
| 1 | Events likely be related to the research and resulting in :<br>- death<br>- immediate life-threatening | SAE initial report form | Within 7 days |
| 2 | Events likely be related to the research and resulting in :<br>- hospitalization<br>- significant disability/incapacity<br><br>*"potentially serious"* events | SAE initial report form | Within 15 days |
| 3 | Death following a previous reported SAE | SAE follow-up report form | Within 7 days |
| 4 | Complementary information on :<br>- diagnosis<br>- initial causal relationship to study drug<br><br>SAE evolution to recovery or aggravation | SAE follow-up report form | Any time |
| 5 | Complementary information required by ANRS | SAE follow-up report form | Within 7 days |

### 1.2.1.

### 1.2.2. PREGNANCIES REPORT AND SAE RELATED TO PREGNANCY

| Case n° | What? | Which form? | When? |
|---|---|---|---|
| 6 | Pregnancy | Pregnancy report form | Within 15 days |
| 7 | Serious pathology occurring during pregnancy resulting in a death or a life threatening condition. | SAE initial report form | Within 7 days |
| 8 | Serious pathology occurring during pregnancy (not in case n°7) | SAE initial report form | Within 15 days |
| 9 | Congenital abnormality | SAE initial report form | Within 15 days |
